# Neuronal FcεRIα directly mediates ocular itch via IgE-immune complex in a mouse model of allergic conjunctivitis

**DOI:** 10.1186/s12974-022-02417-x

**Published:** 2022-02-23

**Authors:** Huan Cui, Fan Liu, Yehong Fang, Tao Wang, Bo Yuan, Chao Ma

**Affiliations:** 1 Department of Human Anatomy, Histology and Embryology, Institute of Basic Medical Sciences, Neuroscience Center, Chinese Academy of Medical Sciences, School of Basic Medicine, Peking Union Medical College, Beijing, China; 2 National Human Brain Bank for Development and Function, Institute of Basic Medical Sciences Chinese Academy of Medical Sciences, School of Basic Medicine Peking Union Medical College, Beijing, China; 3 Chinese Institute for Brain Research, Beijing, China

**Keywords:** Ocular itch, Allergic conjunctivitis, FcεRIα, Sensory neuron

## Abstract

**Background:**

Classical understanding of allergic conjunctivitis (ACJ) suggests that ocular itch results from a mast cell-dependent inflammatory process. However, treatments that target inflammatory mediators or immune cells are often unsatisfying in relieving the stubborn itch symptom. This suggests that additional mechanisms are responsible for ocular itch in ACJ. In this study, we aim to determine the role of neuronal FcεRIa in allergic ocular itch.

**Methods:**

Calcium imaging was applied to observe the effect of IgE-immune complex in trigeminal neurons. Genomic *FcεRIa* knockout mice and adeno-associated virus (AAV) mediated sensory neuron *FcεRIa* knockdown mice were used in conjunction with behavioral tests to determine ocular itch. In addition, immunohistochemistry, Western blot and quantitative RT-PCR were used for in vitro experiments.

**Results:**

We found that FcεRIα was expressed in a subpopulation of conjunctiva sensory neurons. IgE-IC directly activated trigeminal neurons and evoked acute ocular itch without detectible conjunctival inflammation. These effects were attenuated in both a global *FcεRIa*-knockout mice and after sensory neuronal-specific *FcεRIa*-knockdown in the mouse trigeminal ganglion. In an ovalbumin (OVA) induced murine ACJ model, FcεRIα was found upregulated in conjunctiva-innervating CGRP+ sensory neurons. Sensory neuronal-specific knockdown of *FcεRIa* significantly alleviated ocular itch in the ACJ mice without affecting the immune cell infiltration and mast cell activation in conjunctiva. Although FcεRIα mRNA expression was not increased by IgE in dissociated trigeminal ganglion neurons, FcεRIα protein level was enhanced by IgE in a cycloheximide-resistance manner, with concordant enhancement of neuronal responses to IgE-IC. In addition, incremental sensitization gradually enhanced the expression of FcεRIα in small-sized trigeminal neurons and aggravated OVA induced ocular itch.

**Conclusions:**

Our study demonstrates that FcεRIα in pruriceptive neurons directly mediates IgE-IC evoked itch and plays an important role in ocular itch in a mouse model of ACJ. These findings reveal another axis of neuroimmune interaction in allergic itch condition independent to the classical IgE-mast cell pathway, and might suggest novel therapeutic strategies for the treatment of pruritus in ACJ and other immune-related disorders.

**Supplementary Information:**

The online version contains supplementary material available at 10.1186/s12974-022-02417-x.

## Introduction

Allergic conjunctivitis (ACJ) which approximately afflicts 15–20% of the population worldwide actually contains a group of diseases affecting the ocular surface, including seasonal allergic conjunctivitis and perennial allergic conjunctivitis in an acute form, whereas vernal keratoconjunctivitis, atopic keratoconjunctivitis and giant papillary conjunctivitis in a chronic form [1]. Ocular itch is the pathognomonic symptom of ACJ, and significantly reduces the quality of patient's life [2]. Preclinical evidence indicated a direct contribution of IgE-activated mast cells to both the early phase reaction and late-phase inflammation during ACJ [3]. The action of mast cell-driven pruritogens such as histamine and protease to pruriceptors is regarded as the classic peripheral mechanism of itch [4–7]. However, available treatments targeting mast cells and related inflammatory factors such as antihistamine, mast cell stabilizer and steroid are often unsatisfying for ocular itch symptom and/or exhibit adverse side effects [8–10]. Thus, additional mechanisms besides mast cell-associated inflammation probably contribute to ACJ itch, which remain largely unexplored.

The ACJ patients usually have an elevated antigen-specific immunoglobulin (Ig) level, especially IgE in the serum and tears [11, 12]. IgE, a subtype of immunoglobulin associated with allergic diseases, is produced by B cells after immune rearrangement. IgE-immune complex (IgE-IC) formed by allergen and specific IgE can elicit anaphylactic reaction through challenging Fc-epsilon receptors (FcεRs) [13–15]. Fc epsilon receptor I (FcεRI), a high-affinity activating IgE receptor, is defined structurally as a tetrameric complex comprising an α-subunit (FcεRIα, the IgE binding chain), a β-subunit (FcεRIβ), and two γ-subunits (FcεRIγ) [16–19]. FcεRIα plays a central role in allergic diseases through linking IgE and responsive cells, such as mast cells and basophils [15, 20]. β and γ subunits of FcεRI contain conserved immunoreceptor tyrosine-based activation motifs (ITAMs) and transmit intracellular signaling. Omalizumab, the humanized monoclonal IgE antibody, neutralizes the free circulating IgE, thereby preventing the binding of IgE to FcεRI [21]. Clinical evidence showed favorable effects of subcutaneous treatment with omalizumab on antihistamines and mast-cell stabilizers unresponsive Vernal Keratoconjunctivitis, including the ocular itch symptom [22]. Therefore, IgE-FcεRI might give rise to ACJ itch in a mast cell and inflammation independent manner.

Although IgE-FcεRI signaling has been suggested to serve as a critical element in ACJ pathogenesis, its potential role in ocular itch under ACJ conditions remains unclear. Moreover, the FcεRI is also expressed in peripheral sensory neurons besides the immune cells, such as mast cells and basophils. This neuronal receptor is functional, since antigen application could directly induce neuronal activation after sensitized with antigen-specific IgE [23, 24]. According to our previous research, IgE-IC also activated a subpopulation of trigeminal ganglion (TG) neurons and FcεRI might participate in allergic ocular itch based on pharmacological results [25]. However, the in vivo evidence that neuronal FcεRI contributes to ocular itch has not been explored. No studies have addressed whether FcεRI is expressed in conjunctiva sensory neurons or whether IgE-IC acts directly on conjunctiva pruriceptors through neuronal FcεRI to induce ocular itch. In this study, we tested the hypothesis that IgE-IC contributes to ACJ conjunctiva itch through direct activation of neuronal FcεRI.

## Materials and methods

### Animals

Adult male mice (20–25 g; 6–8 weeks) were used in this study. Mice were housed under a 12-h light/12-h dark cycle with ad libitum access to food and water. Wild type C57BL/6 mice were purchased from the National Institutes for Food and Drug Control in China. The *c-Kit *^*W-sh/W-sh*^, *Mrgpra3*^*GFP-cre*^, and *Mrgpra3*^*GFP-Cre*^; *ROSA26*^*tdTomato*^ mice were provided by Dr. Xinzhong Dong of Johns Hopkins University [26, 27]. The *Fcer1a*-deficient mice (*Fcer1a*^*−/−*^) were provided by Jing Wang of Institute of Basic Medical Sciences, Chinese Academy of Medical Sciences, Department of Pathophysiology, Peking Union Medical College [28].

### Sensitization and challenging of mice

The construction of the ACJ model was performed following our previous study. Briefly, on day 0, day 7, and day 14, i.p. injection of 100 μg ovalbumin (OVA, Sigma-Aldrich, St. Louis, MO, USA) plus 100 μl Imject Alum (Thermo Scientific, Rockford, IL, USA) dissolved in 200 μl of PBS. On day 21, 5 μl of OVA (1% in PBS) was applied topically to the lower conjunctival sac of both sides to induce ocular allergy. For the control-treated mice, OVA was topically applied in the lower conjunctival sac without any sensitization. The video record was applied immediately as OVA instillation, and tissue collection was performed approximately 12 h after the OVA challenge.

For the incremental sensitization workflow, the mice were divided into four groups: Control, Sensitized 1w, Sensitized 2w, and Sensitized 3w, respectively. Sensitization was carried out as following procedures: For Sensitized 3w group, on day 0, day 7, and day 14, i.p. injection of 100 μg OVA (Sigma-Aldrich, St. Louis, MO, USA) plus 100 μl Imject Alum (Thermo Scientific, Rockford, IL, USA) dissolved in 200 μl of PBS. Sensitized 1w and Sensitized 2w groups mice received sensitization once on day 0 and twice on day 0 and day 7, respectively, while Control group did not receive any sensitization. The mice used for immunohistochemistry and Western blot were decapitated and taken the TG tissues 1 week after the last sensitization without challenge. For the behavioral test, 1% OVA in 5 μl PBS or 5 μl PBS was applied topically to both eyes to induce allergic conjunctivitis 1 week after the last sensitization. The operators for the above experiments were double-blind.

### Behavioral assays

Behavioral tests for ocular itch were carried out following the methods described in previous publications [25, 29, 30]. Mice were placed in acrylic chambers (13 × 9 × 40 cm) in a sound-proof room without persons for 3 consecutive days to allow acclimation. For the acute itch model, the tested mice were manually restrained, and 5 µl of regents (capsaicin, 5 μM in PBS containing 0.1% Tween 20; histamine; IgE-IC, 1–50 µg/ml in 5 µl PBS; IgE, 50 µg/ml in 5 µl PBS; OVA, 100 mg/ml in 5 µl PBS) were applied gently into the inferior conjunctival sac. Then, mice were returned to recording chambers and recorded with a high-resolution digital camera (SONY HANDYCAM HDR-PJ580E, Japan) for 15 min or 1 h. A single scratching bout was defined as the animal lifting its hind paw, scratching the treated eyes. A wiping bout was defined as the animal lifting its forelimb, wiping the ocular area, and returning to its original start position [30].

For the sensitized mice, the OVA challenge was performed 7 days after the last sensitization. 1% OVA in 5 µl PBS was gently instilled into the inferior conjunctival sac. Afterwards, the challenged mice were immediately returned back to the recording chambers to film for 1 h. The scratching and wiping bouts were separately counted as scored the video-recorded behavior. Each applied regent was previously prepared and then coded by a laboratory assistant and not the experimenter. The experimenter who injected the chemical was blinded to the code and thus the chemical injected, as was the observer who scored the video-recorded behavior.

### Retrograde labeling

Wheat Germ Agglutinin (Alexa Fluor™ 350 Conjugated WGA, Thermo Fisher Scientific, W11263; 2 μl in PBS) was injected into the submucosa of the palpebral conjunctiva using an insulin syringe as anesthetized by isoflurane [29]. Tissue collections were performed 48–60 h after the injection.

### Preparation of IgE-immune complex

IgE-IC was formed by incubating OVA (48 μg/ml) (Sigma-Aldrich, St. Louis, MO, USA) and mouse anti-OVA IgE (80 μg/ml) (Acris Antibodies, Inc, Rockville, MD, USA) for one h at 37 °C, and the IgE-IC was diluted to 1 μg/ml, 10 μg/ml, 50 μg/ml for the behavioral test. Meanwhile, IgE-IC was diluted to 0.1 μg/ml for calcium imaging use. The ratio of OVA and mouse anti-OVA IgE was 2:1. The storage buffer of all the antibodies was exchanged to PBS buffer using Zeba™ spin desalting columns (Thermo Scientific, Rockford, IL, USA) before application to avoid the possible toxic or non-specific effects of sodium azide [25].

### Trigeminal injection

The trigeminal injection was performed following previous studies [31, 32]. The shRNA targeting the sequence of mice *FcεRIa* (Gene Bank Accession: NM_14125, sh*FcεRIa*) or additional scrambled sequence (Negative control, NC) was designed, respectively. Pirt promoter (− 2000 to + 200 bp) was applied to guide the expression of shRNA in trigeminal sensory neurons. The recombinant adeno-associated virus type 9 (AAV9) containing Pirt promoter-guided sh*FcεRIa* sequence (AAV9-pirt-shFcεRIa*-*EGFP) or Pirt promoter-guided NC sequence (AAV9-pirt-NC-EGFP) was packaged using EGFP vector. The relative sequences are: *shFcεRIa*, 5′-GCUAUGGGAACAAUCACCUUCAAAU-3′ and NC, 5′-TTC TCC GAA CGT GTC ACG T-3′. The adeno-associated virus (1 × 10^12^ TU, 1 μl) was injected into the trigeminal ganglion through the infraorbital foramen using a 30 G needle. Two weeks after trigeminal injection, mice were used for relative experiments.

### Cell dissociation and culture

Calcium imaging of cultured trigeminal ganglion neurons was performed as described previously [25]. TGs were harvested and transferred into the oxygenated complete saline solution (CSS) for cleaning and mincing. The TGs were then digested with Liberase TM (Roche, Basel, Switzerland) for 20 min and for another 10 min with Liberase TL (Roche, Basel, Switzerland) and papain (30U/ml; Worthington Biochemical, Lakewood, NJ, USA) in CSS containing 0.5 mM EDTA at 37 °C. After enzymatic digestion, the cells were dissociated by gentle trituration with a fire-polished Pasteur pipette in a culture medium containing 0.5 mg/ml bovine serum albumin and 0.5 mg/ml trypsin inhibitor and placed on poly-d-lysine/laminin-coated 12-mm diameter circular glass coverslips. The culture medium contained equal amounts of DMEM and F12 with 10% FBS. The cells were maintained at 37 °C in a humidified atmosphere of 95% air and 5% CO_2_ and were used within 24 h. Isotype IgE (ab37425, abcam) was added to the medium for 24 h to treat the dissociated neurons before the extraction of RNA or protein. Cycloheximide (10 μg/ml, HY-12320, MedChemExpress), a potent inhibitor of translation, was added to the culture medium and treated the dissociated neurons for 24 h with or without IgE (5 μg/ml).

### Intracellular calcium imaging

Intracellular calcium imaging was performed as described before [25]. Briefly, after loading Fura 2-acetoxy-methyl ester (2 μM) in the dark for 45 min at 37 °C, TG neurons were placed in a recording chamber continuously perfused with HEPES buffer at a flow rate of 1.5 ml/min at room temperature.

All reagents were dissolved in HEPES buffer and applied locally to the neuronal cell bodies through a micropipette (with a tip diameter of 100 μm). For the *Mrgpra3*^*GFP-Cre*^ mouse line, we carried out the procedure to the cells that we detected GFP by fluorescence microscope. HEPES containing 1 μM capsaicin or 50 mM K^+^ was used to confirm the responses to capsaicin and viability of neurons at the end of each experiment. Neurons were categorized according to the diameter of soma as small- (< 25 μm), medium- (25–35 μm) and large-sized (> 35 μm).

### Immunohistochemistry

Trigeminal ganglions from a male donor were obtained from the Brain Bank of Chinese Academy of Medical Science & Peking Union Medical College, which had got informed consent for using the donated body tissue for medical research. The trigeminal ganglions were fixed in 10% formalin and then cryoprotected in 30% sucrose overnight. The tissue was sectioned at 10 μm thick on a cryostat for the next immunohistochemistry experiment.

For mice receiving IgE-IC instillation, tissue collection was performed one after IgE-IC application, while tissue collection was performed approximately 12 h after OVA instillation for the ACJ model. All mice used for histology were anesthetized with pentobarbital sodium and transcardially perfused with ice-cold PBS followed by ice-cold 4% PFA [29]. Tissues were post-fixed in ice-cold 4% PFA (6 h for conjunctiva; 1 h for TGs; 2 h for spleen) and cryoprotected in 30% (w/v) sucrose for 24 h before they were embedded and frozen in OCT compound. The TGs and conjunctivas were sectioned at 10 μm thick on a cryostat. After incubated with 10% normal horse serum for 1 h, tissue sections were incubated at 4 °C overnight with primary antibodies. After being washed with PBS, sections were incubated with corresponding secondary antibodies for 1 h at room temperature. All antibodies used for immunohistochemistry are listed in Additional file 2: Table S1. For mast cell staining, tissue sections were incubated with FITC-conjugated avidin (Thermo Fisher Scientific, 434411) for 15 min at room temperature. After staining, tissue sections were mounted using fluorescent mounting medium (ZSGB-BIO, ZLI-9556, and ZLI-9557) and imaged after drying. Images were captured by a laser confocal microscopic imaging system (Olympus FV1000 and FluoView software). Neurons were classified as small- (area < 442 µm^2^), medium- (area 443–865 µm^2^), and large-sized (area > 865 µm^2^) according to their cross-sectional areas [33].

### Western blot

Mouse TG tissues or dissociated TG neurons (cultured 24 h) were harvested and homogenized in RIPA buffer (CW-bio, Beijing, China) with protease inhibitors (CW-bio, Beijing, China). The homogenates were separated by SDS–PAGE gel and transferred to PVDF membranes. The membranes were incubated at 4 °C overnight by primary antibody (rabbit anti-FcεRIα, 1:1000 and mouse anti-β-actin, 1:2000) following incubated by 5% BSA for 1 h. After incubated with the corresponding HRP-conjugated anti-IgG antibody (goat anti-rabbit IgG, 1:3000 and goat anti-mouse IgG, 1:3000), bands were determined using eECL Kit.

### Quantitative RT-PCR

Total RNAs of cultured TG neurons were extracted by Trizol reagent (CW-bio, Beijing, China), which were reversely transcribed by RT Master Mix (Takara, Japan). qRT-PCR was performed using a CFX96™ Real-Time PCR Detection System (Bio-Rad, Hercules, California, USA) with SYBR premix Ex Taq™ (Takara, Japan). The primers used were as follow: FcεRIα Forward, 5′-GCT ATG GGA ACA ATC ACC TTC A-3′ and Revers 5′-GGC ACT CAC AAT GAC CAA ATG T-3′; β-Actin Forward, 5′-GTC CCT CAC CCT CCC AAA AG-3′ and Revers 5′-GCT GCC TCA ACA CCT CAA CCC-3′.

### Statistical analysis

Data values were presented as means with standard errors (mean ± SEM). Statistical analyses were performed using the SPSS software (version 17.0). A Student′s *t* test was used to evaluate the statistical significance of a difference between two groups. Comparisons for multiple groups or multiple timepoints were carried out using a 1-way or 2-way ANOVA for random measures or repeated measures followed by Bonferroni’s post hoc test comparisons. Chi-square tests were used to compare between two or more incidence of events. The criterion for statistical significance was a value of *p* < 0.05.

## Results

### FcεRIα is expressed in mouse conjunctiva-innervating TG neurons

**Fig. 1:**
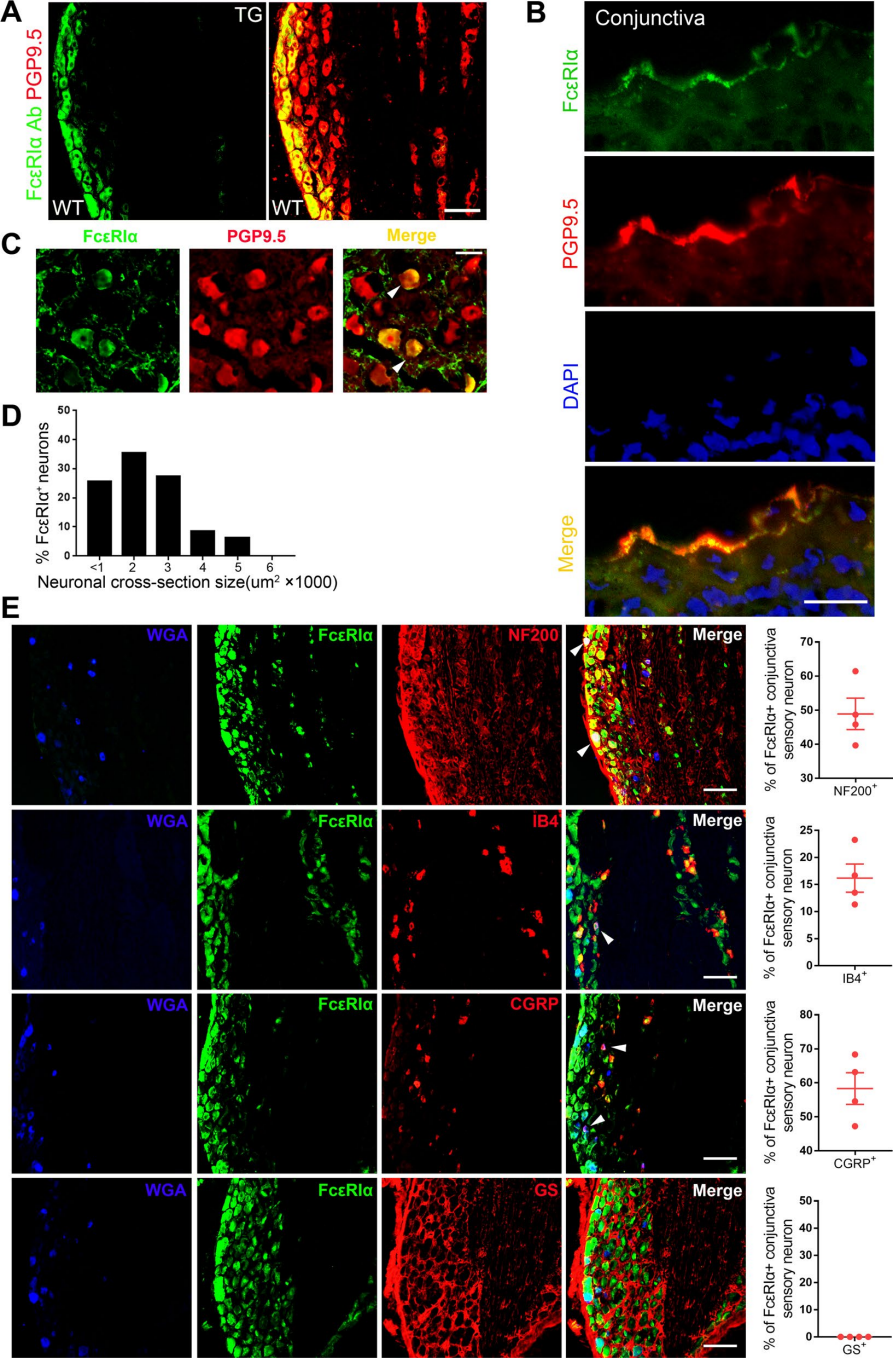
Analysis of FcεRIα expression in conjunctiva-innervating sensory neurons. **A** Double-immunostaining showed that FcεRIα signal was colocalized with the neuronal marker PGP9.5 in the trigeminal ganglion of mice. Scale bar: 100 μm. **B** FcεRIα was detected in PGP9.5^+^ neuronal fiber in the conjunctiva of mice. Scale bar: 25 μm. **C** Double-immunostaining showed that FcεRIα signal was colocalized with the neuronal marker PGP9.5 in the trigeminal ganglion of human. Scale bar: 100 μm. **D** Expression pattern of FcεRIα along different sizes trigeminal neurons in human trigeminal ganglion. **E** Fluorescent immunostaining in WGA-labeled (blue) TG for FcεRIα (green) and markers (red) including NF200 (*n* = 4 mice), IB4 (*n* = 4 mice), CGRP (*n* = 4 mice), and glutamine synthetase (GS; *n* = 4 mice), along with quantitative analysis of percentage overlap. Scale bars: 100 μm

First, immunofluorescence (IF) results showed that FcεRIα was colocalized with neuronal marker PGP9.5 in trigeminal ganglion (TG), suggesting the expression of FcεRIα in sensory neurons (Fig. 1A). Meanwhile, FcεRIα expression was also detected in the PGP9.5-positive conjunctival nerve fiber (Fig. 1B). The effectiveness of the FcεRIα antibody was validated by the lost IF signal in the TG and spleen of global *FcεRIα*^*–/–*^ mice. In addition, the specificity of the FcεRIα antibody was verified by the lost IF signal in the TG as detected by the isotype IgG (Additional file 1: Fig. S1A–C). From a human TG, we found that FcεRIα was expressed in sensory neurons with a percentage of 25.80% (89/345) (Fig. 1C, D). Furthermore, we performed double immunostaining to map the expression pattern of FcεRIα in conjunctiva-innervating TG neurons that WGA retrogradely labeled. Among WGA-labeled conjunctiva sensory neurons, 48.91% of FcεRIα^+^ neurons coexpressed NF200, a marker for large-diameter neurons. In addition, 16.19% of FcεRIα^+^ neurons coexpressed IB4, a nonpeptidergic nociceptive marker. Notably, 58.31% of FcεRIα^+^ neurons coexpressed CGRP, a peptidergic nociceptive marker. However, no obvious colocalization of FcεRIα protein expression with glutamine synthetase, a satellite glial cell marker, was detected (Fig. 1E). Using *Mrgpra3*^*GFP-Cre*^ line, FcεRIα-positive trigeminal neurons colocalized with MrgprA3, a pruriceptor marker (Additional file 1: Fig. S1D, E). Moreover, the FcεRIα signal was also detected in MrpgrA3^+^ conjunctival nerve fiber of *Mrgpra3*^*GFP-Cre*^; *ROSA26*^*tdTomato*^ mice (Additional file 1: Fig. S1F–H). These results imply that a subpopulation of conjunctiva sensory neurons, including nociceptors and pruriceptor, express FcεRIα, providing an anatomical basis for FcεRIα-mediated conjunctiva itch.

### IgE-IC directly activates TG neurons through FcεRIα

**Fig. 2:**
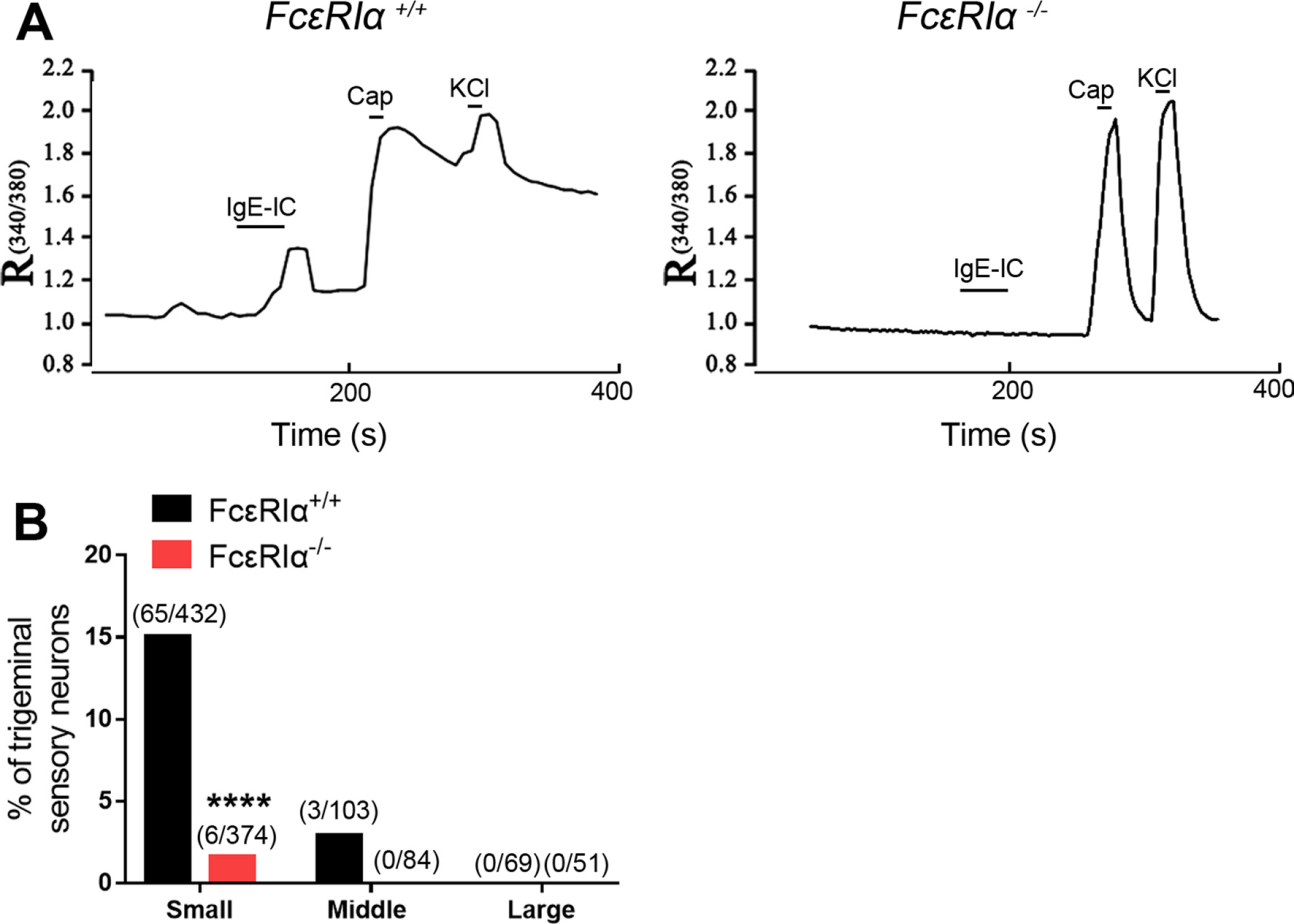
IgE-IC directly activates trigeminal sensory neurons through FcεRIα in vitro. **A** Representative traces of Ca^2+^ responses induced by IgE-IC (0.1 μg/ml, 30 s), capsaicin (Cap; 1 μM, 10 s), and KCl (50 mM, 10 s) in trigeminal neurons from *FcεRIα*^+*/*+^ (left) and global *FcεRIα*^*–/–*^ (right) mice. **B** Quantitative analysis showed that IgE-IC evoked Ca 2^+^ responses in a larger proportion of small-diameter (< 25 μm) trigeminal neurons from FcεRIα^+/+^ mice than those from *FcεRIα*^*–/–*^ mice. ****P < *0.0001* vs. *FcεRIα*^+*/*+^, χ^2^ test. No significance was detected between *FcεRIα*^+*/*+^ mice and *FcεRIα*^*–/–*^ mice for middle- (25–35 μm) and large-diameter (> 35 μm) trigeminal neurons

To identify the effects of FcεRIα expressed in TG sensory neurons on Ca^2+^ responses evoked by IgE-IC, we applied Ca^2+^ imaging in dissociated TG neurons from WT (*Fcer1a*^+/+^) and global FcεRIα knockout (*Fcer1a*^*−/−*^) mice. We observed that IgE-IC responsive subpopulation was mainly small-diameter neurons (65/68, 95.59%) in *Fcer1a*^+/+^ mice, one special character of nociceptors. In *Fcer1a*^+/+^ mice, application of IgE-IC (1 μg/ml) evoked Ca^2+^ increases in 15.05% of small-diameter TG sensory neurons, the majority of which also reacted to capsaicin. Nevertheless, IgE-IC evoked Ca^2+^ responses in a significantly smaller fraction (1.60%) of small-diameter TG neurons from *Fcer1a*^*−/−*^ mice (Fig. 2). Using *Mrgpra3*^*GFP-Cre*^; *ROSA26*^*tdTomato*^ mice, we detected Ca^2+^ responses in MrgprA3^+^ neuron (24/98, 24.49%) as stimulated by IgE-IC (1 μg/ml) (Additional file 1: Fig. S2). The above findings indicate that IgE-IC can directly activate TG sensory neurons in a FcεRIα-depended manner, which might be involved in ocular itch.

### Local instillation of IgE-IC elicits acute ocular itch without obvious inflammation in naive mice

**Fig. 3:**
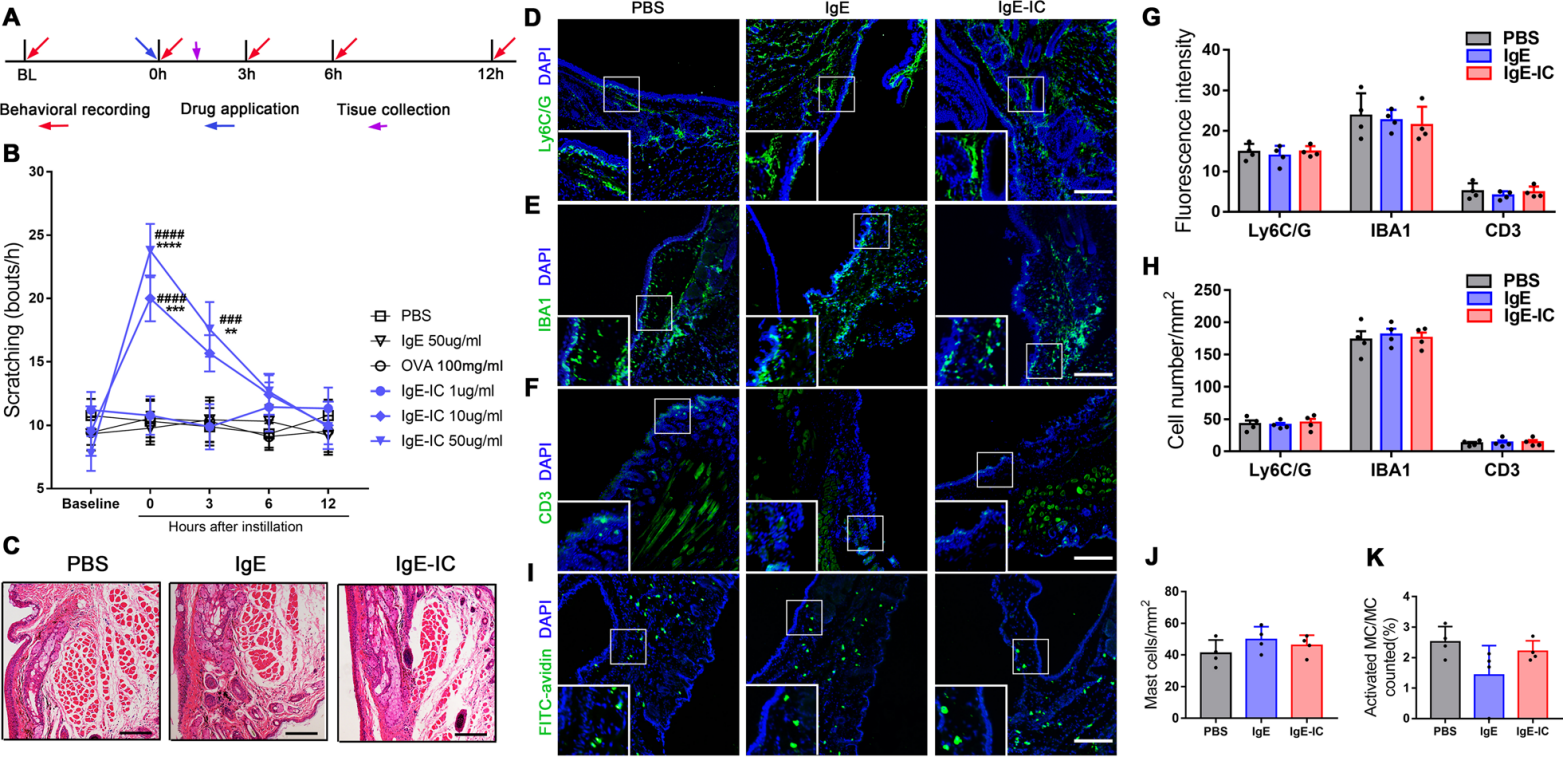
IgE-IC elicits acute ocular itch in naive mice. **A** Timeline of behavioral recording, drug application, and tissue collection. **B** Mice were ocular instilled with IgE-IC (1, 10, 50 μg/ml; 5 μl), monomeric IgE (50 μg/ml; 5 μl), OVA (100 mg/ml, 5 μl) or vehicle (PBS; 5 μl), and eye-towards scratching bouts were counted over 1–12 h. Baseline was identified as recorded without any instillation. n = 8–10 mice per group; **P < *0.01* vs. PBS, ***P < *0.001* vs. PBS, ****P < *0.0001* vs. PBS; ^###^*P* < *0.001* vs. Baseline, ^####^*P* < *0.0001* vs. Baseline; 2-way ANOVA for repeated measures followed by Bonferroni’s post hoc test. **C** Representative images of conjunctiva taken 1 h after ocular instillation with PBS, monomeric IgE (50 μg/ml), or IgE-IC (50 μg/ml), stained with H&E. Scale bar: 100 μm. **D**–**F** Representative images of conjunctiva taken 1 h after ocular instillation with either PBS, monomeric IgE (50 μg/ml), or IgE-IC (50 μg/ml) and stained for Ly6C/G (**D**), IBA1 (**E**), and CD3 (**F**). Scale bar: 100 μm. The embedded boxes showed the enlarged views. **G** Quantification for fluorescence intensity of markers showed no significant differences between treatment groups. *n* = 4 per group; one-way ANOVA followed by Bonferroni’s post hoc test comparisons. **H** Quantitative analysis of Ly6C/G+, IBA+, and CD3+ cell numbers after different treatments. *n* = 4 per group; one-way ANOVA followed by Bonferroni’s post hoc test comparisons. **I** Representative images of conjunctiva taken 1 h after ocular instillation with either PBS, monomeric IgE (50 μg/ml), or IgE-IC (50 μg/ml) and stained for FITC-avidin. Scale bar: 100 μm. The embedded boxes showed the enlarged views. **J** Quantitative analysis of mast cells number after different treatments. *n* = 4 per group; one-way ANOVA followed by Bonferroni’s post hoc test comparisons. **K** Proportions of activated mast cells (with degranulation) after different treatments. *n* = 4 per group; one-way ANOVA followed by Bonferroni’s post hoc test comparisons

Since IgE-IC is able to stimulate immune cells and directly activate sensory neurons, IgE-IC was locally instilled in the conjunctiva to assess its effects on the inflammatory process and ocular itch (Fig. 3A). Ocular instillation of IgE-IC (10 µg/ml and 50 µg/ml), but not the vehicle (PBS), antigen (100 mg/ml OVA in PBS), or monomeric IgE, immediately induced eye-towards scratching behavior, one indicator for the ocular itch. In addition, this ocular itch could last to 3 h after IgE-IC application but could not be detected from 6 h (Fig. 3B). However, no significant difference was detected for the eye-towards wiping behavior, one indicator for ocular pain (Additional file 1: Fig. S3A). The H&E staining for the conjunctivas did not detect any signs of immune cell infiltration at 1 h after instillation (Fig. 3C). To further investigate the effects of ocular instillation of IgE-IC on local immune status, we used immunofluorescence staining to assess cellular infiltration of neutrophils (marked by Ly6C/G), macrophages (marked by IBA1), and lymphocytes (marked by CD3) into the conjunctiva. One hour after ocular instillation, no significant differences were observed in any of these markers for mice receiving PBS, IgE, and IgE-IC treatment (Fig. 3D–H). We next examined the effects of IgE-IC instillation on infiltration and activation of mast cells. The numbers of mast cells within the conjunctiva sections (labeled by FITC-avidin) showed no difference in the IgE-IC instillation group compared with PBS and monomeric IgE. Meanwhile, the percentages of degranulated mast cells also showed no difference for the mice receiving IgE-IC instillation (Fig. 3I–K). In addition, we did not observe obvious immune cell infiltration (neutrophils, macrophages, and lymphocytes) within TG 1 h after instillation (Additional file 1: Fig. S3B–E). We detected mast cells within the TG tissue, while no obvious mast cells infiltration and activation were noted in TG from mice treated by PBS, IgE, and IgE-IC (Additional file 1: Fig. S3F–H). These results suggest that IgG-IC is sufficient to specifically evoke behavioral signs of acute ocular itch, but not pain, without detective immune cell infiltration and mast cell activation in conjunctiva and TG, at least at the early stages.

### FcεRIα mediated acute ocular itch induced by IgE-IC

**Fig. 4:**
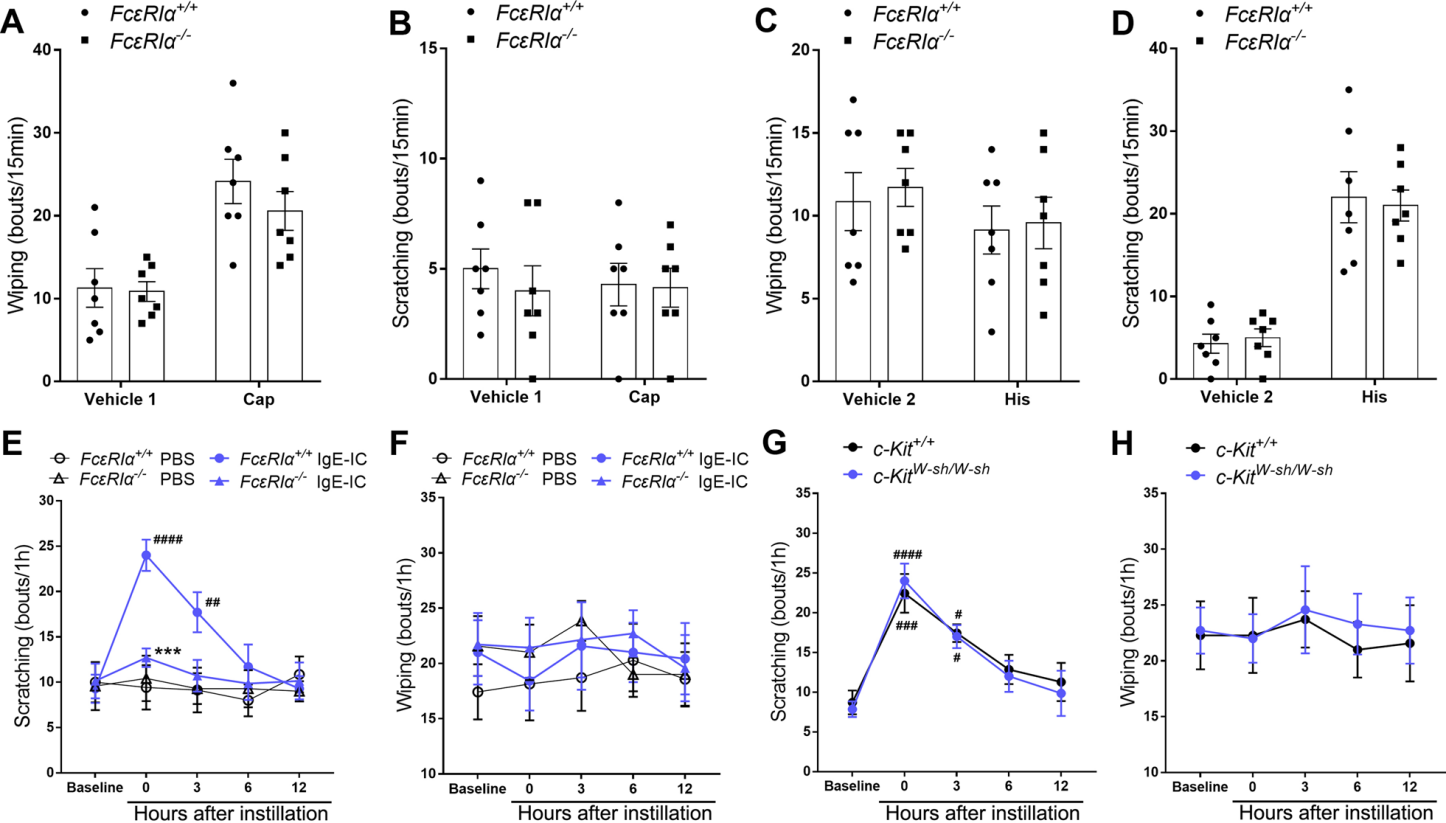
IgE-IC induces acute ocular itch in a FcεRIα-dependent manner. **A**, **B** Eye-towards wiping (**A**) and scratching (**B**) response of *FcεRIα*^+*/*+^ and *FcεRIα*^*−/−*^ mice as ocular application of capsaicin (Cap). No difference was detected for *FcεRIα*^+*/*+^ and *FcεRIα*^*−/−*^ mice for both reaction to Vehicle 1 and Cap. *n* = 7, Student’s *t*-test. **C**, **D** Eye-towards wiping (**C**) and scratching (**D**) response of *FcεRIα*^+*/*+^ and *FcεRIα*^*−/−*^ mice as ocular application of histamine (His). No difference was detected for *FcεRIα*^+*/*+^ and *FcεRIα*^*−/−*^ mice for both reaction to Vehicle 2 and His. *n* = 7, *t* test. **E**, **F** Eye-towards scratching (**E**) and wiping (**F**) response of *FcεRIα*^+*/*+^ and *FcεRIα*^*−/−*^ mice as ocular instillation of IgE-IC or PBS. ^##^*P* < *0.01*, ^###^*P* < *0.001*, IgE-IC vs. PBS; ***P < *0.001*, *FcεRIα*^+*/*+^ vs. *FcεRIα*^*−/−*^; Two-way ANOVA followed by Bonferroni’s post hoc test comparisons. **G**, **H** Eye-towards scratching (**G**) and wiping (**H**) response of *c-Kit*^+*/*+^ and *c-Kit *^*W-sh/W-sh*^ mice as ocular instillation of IgE-IC. *n* = 7, ^#^*P* < *0.05*, ^###^*P* < *0.001*, ^####^*P* < *0.0001*, vs. Baseline, two-way ANOVA followed by Bonferroni’s post hoc test comparisons

Since FcεRIα is the subunit combining IgE-IC, we applied global FcεRIα knockout (*FcεRIα*^*–/–*^) mice to determine whether FcεRIα mediates the acute ocular itch induced by IgE-IC. The *FcεRIα*^*–/–*^ mice exhibited normal pain-like ocular reaction (wiping) to the algesic substance (capsaicin) compared with *FcεRIα*^+*/*+^ mice (Fig. 4A, B). In addition, pruritogens (histamine) could induce normal itch-like ocular reaction (scratching) in *FcεRIα*^*–/–*^ mice compared with *FcεRIα*^+*/*+^ mice (Fig. 4C, D). However, the ocular scratching responses to conjunctival instillation of IgE-IC were significantly attenuated in *FcεRIα*^*–/–*^ mice compared with *FcεRIα*^+*/*+^ mice (Fig. 4E, F). These findings suggest that FcεRIα is necessary for IgE-IC-elicited acute ocular itch. Given that FcεRIα was also expressed in mast cells, we next investigated whether mast cells were required for IgE-IC-induced acute ocular itch. In the mast cell-deficient *c-Kit *^*W-sh/W-sh*^ mouse line, we observed no significant differences in basal or in IgE-IC-evoked ocular scratching behavior compared with WT controls (Fig. 4G, H). Together, these results indicate that the acute itch effects of IgE-IC are mediated by FcεRIα, but do not specifically require mast cells, which suggests that IgE-IC might directly combine FcεRIα in conjunctiva-innervating TG neurons to elicit itch sensation.

### Neuronal FcεRIα mediated acute ocular itch induced by IgE-IC

**Fig. 5:**
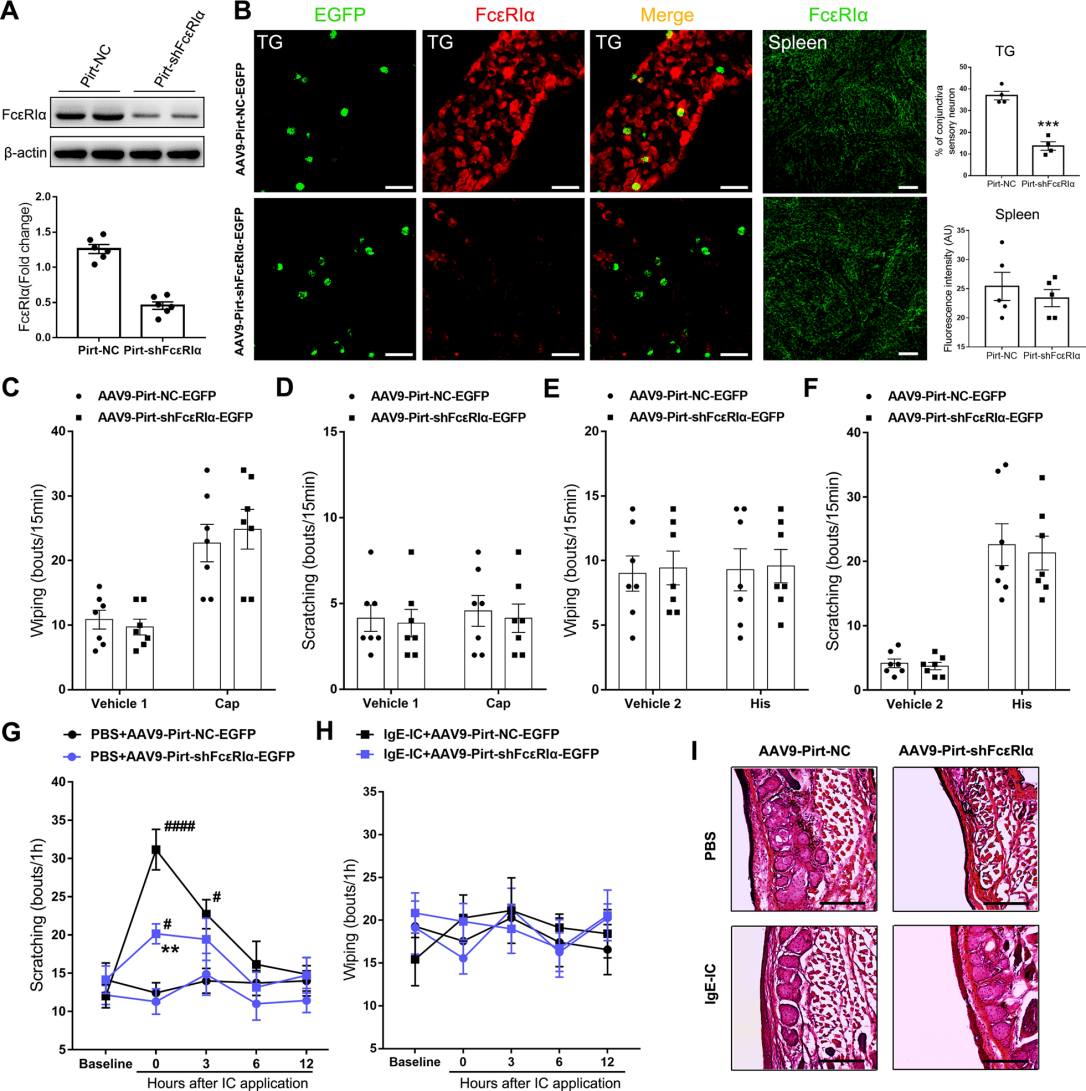
Neuronal FcεRIα mediates acute ocular itch induced by IgE-IC. **A** Representative gel band of trigeminal FcεRIα protein from mice injected AAV9-Pirt-NC-EGFP and AAV9-Pirt-shFcεRIα-EGFP. Western blot revealed a significant decrease in the protein level of FcεRIα in TG infected by AAV9-Pirt-shFcεRIα-EGFP compared with AAV9-Pirt-NC-EGFP. *n* = 4 mice/group, Student’s *t*-test. **B** Representative images of EGFP signal and FcεRIα labeled by immunostaining in TG from mice infected by viruses. Scale bar: 50 μm. Quantification showed reduced FcεRIα expression in TG but not in spleen of mice infected by AAV9-Pirt-shFcεRIα-EGFP compared with those infected by AAV9-Pirt-NC-EGFP. *n* = 4 mice per group; ***P < *0.001*, Student’s *t*-test. **C**, **F** No significant differences were observed between mice infected by two viruses in pain-related wiping behavior and itch-related scratching behavior as instilled with capsaicin (**C**, **D**) or histamine (**E**, **F**). *n* = 7 mice per group; *P* > 0.05, Student’s *t*-test. **G**, **H** Time course of eye-towards scratching (**G**) and wiping (**H**) behavior before and after ocular instillation of IgE-IC (50 μg/ml; 5 μl) in mice infected by two designed viruses. ^#^*P* < *0.05*, ^###^*P* < *0.001*, IgE-IC vs. PBS within same virus; **P < *0.01*, compared between two viruses as treated by IgE-IC. **I** Representative H&E images of conjunctiva from mice infected by two types viruses taken 1 h after ocular instillation with PBS or IgE-IC (50 μg/ml). Scale bar: 100 μm

To more specifically identify the role of neuronal FcεRIα in the IgE-IC-induced ocular itch, we applied AAV-mediated FcεRIα-knockdown under the control of Pirt promoter (AAV9-Pirt-shFcεRIα-EGFP), a strong and selective pan-DRG and TG promoter. Western blot analysis validated the effectiveness of shFcεRIα sequence-loaded virus which interfered with trigeminal FcεRIα expression compared with NC sequence-loaded virus (AAV9-Pirt-NC-EGFP) (Fig. 5A). In addition, we detected the EGFP signal 14 days after the intra-TG injection together with decreased expression of FcεRIα trigeminal neurons in the AAV9-pirt-shFcεRIα-EGFP group. Nevertheless, expression of FcεRIα in the spleen presented no difference for mice receiving the intr-TG injection of these two types viruses (Fig. 5B). To check the normality of acute pain and itch reaction, we applied ocular instillation of capsaicin and histamine to mice infected by these two viruses. Both the wiping reaction to capsaicin and the scratching reaction to histamine showed no difference between these two groups, indicating that neuronal FcεRIα-knockdown did not alter the baseline nociception (Fig. 5C–F). As instilled IgE-IC (50 µg/ml) into the inferior conjunctival sac, mice infected by the control virus presented scratching behavior directed at the treated conjunctiva, which could last to 3 h after instillation. Infected by the shFcεRIα-loaded virus, mice exhibited immediate scratching reaction as stimulated by IgE-IC, whereas the degree was significantly decreased compared with the control virus group. Moreover, no obvious itch reaction was detected for mice infected by shFcεRIα-loaded virus 3 h after IgE-IC application compared with PBS (Fig. 5G). In addition, the pain-related wiping reaction could not be evoked by IgE-IC for mice infected by both two types of viruses (Fig. 5H). The H&E staining for the conjunctivas did not detect any signs of conjunctival inflammation at 1 h after instillation (Fig. 5I). The above findings suggest that IgE-IC evokes acute ocular itch, at least partly, through neuronal FcεRIα.

### ACJ upregulated the expression of FcεRIα in TG innervating the conjunctiva

**Fig. 6:**
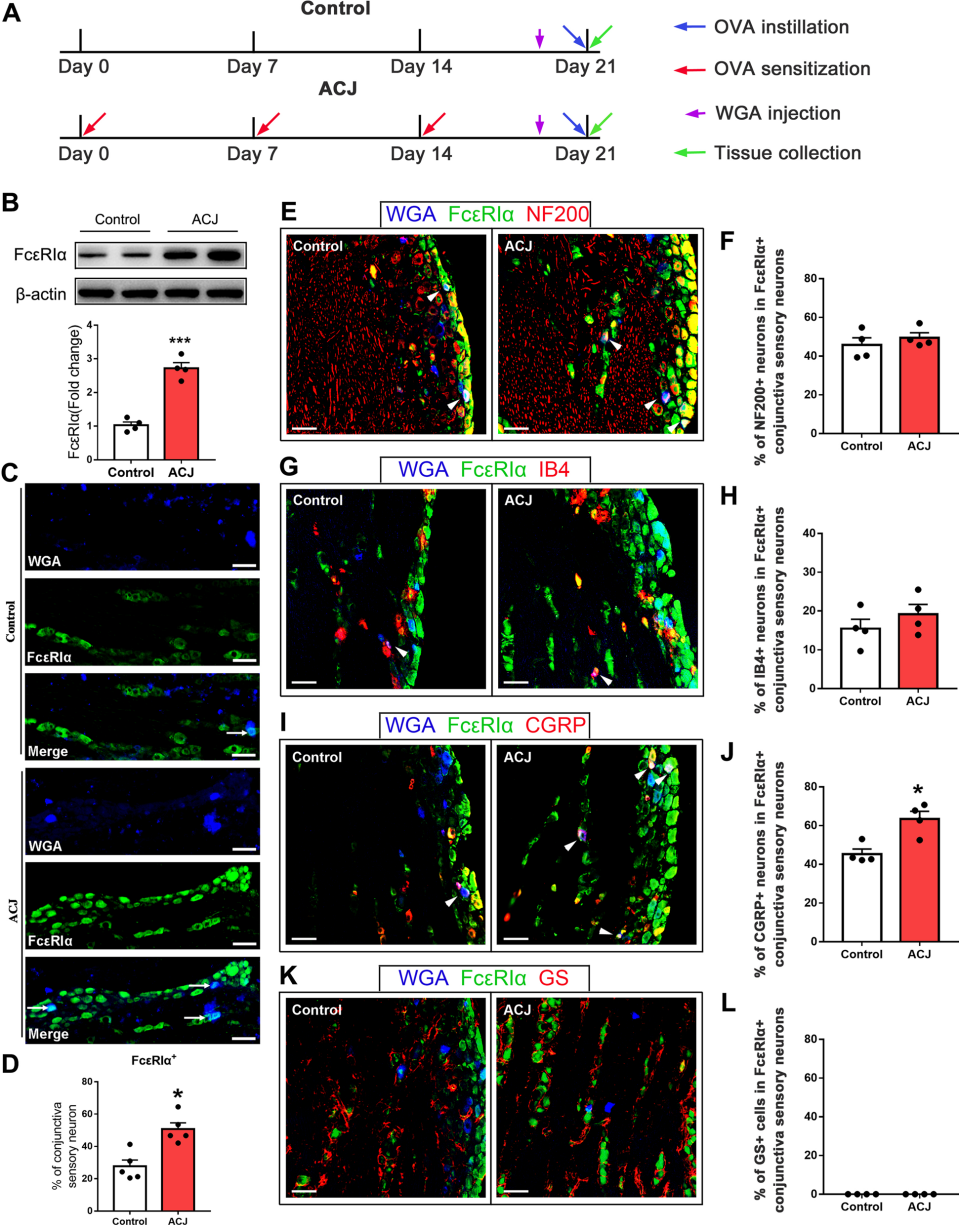
ACJ upregulates the expression of FcεRIα in mouse TG neurons innervating conjunctiva. **A** Schematic diagram of ACJ model construction and workflow. **B** Representative gel band of FcεRIα protein in TG from control and ACJ mice. Western blot revealed a significant increase in the level of FcεRIα protein in TG. *n* = 4 mice/group, ***P < *0.001*, ACJ vs. Control mice, Student’s *t*-test. **C** Typical microscopic images of immunofluorescence staining for FcεRIα in TG sections retrogradely labeled by conjunctival wheat germ agglutinin (WGA) from Control and ACJ mice. **D** ACJ upregulated FcεRIα+ among WGA-labeled conjunctiva sensory neuron. Each point represents data from one mouse, *n* = 5 mice/group, *P < *0.05*, Student’s *t*-test. Scale bar: 50 μm. **E**–**L**. Representative images of TG immunofluorescence staining for FcεRIα and NF200 (**E**), IB4 (**G**), CGRP (**I**) and GS (**K**) in sections containing sensory neurons retrogradely labeled by conjunctival WGA from Control and ACJ mice. Percentage of NF200^+^ (**F**), IB4^+^ (**H**), CGRP^+^ (**J**), and GS^+^ (**L**) markers among FcεRIα^+^ conjunctiva sensory neurons in Control and ACJ mice was summarized. Each point in **F**, **H**, **J**, and **L** represented data from one mouse, *n* = 4 mice/group, *P < *0.05*, Student’s *t*-test. Scale bar: 50 μm

To observe the neuronal expression of FcεRIα under allergic conditions, we produced an OVA-mediated allergic conjunctivitis (ACJ) murine model (Fig. 6A). Western blot results showed that the protein level of trigeminal FcεRIα was significantly upregulated in ACJ mice compared with the vehicle control-treated animals (Fig. 6B). To specifically compare the expression of FcεRIα in conjunctival sensory neurons, we injected WGA into conjunctiva for control and ACJ mice and detected FcεRIα within WGA-labeled trigeminal neuron. The immunofluorescence results showed that the percentage of WGA-labeled FcεRIα^+^ neurons was significantly increased in ACJ mice (Fig. 6C, D). To further identify the character of these conjunctival FcεRIα^+^ sensory neurons, we applied double immunostaining for FcεRIα and a series of markers. Among these FcεRIα^+^ conjunctiva sensory neurons, no significant difference was detected in NF200 and IB4 markers in ACJ mice as compared with the control-treated mice (Fig. 6E–H). However, an elevated proportion of FcεRIα^+^ conjunctiva sensory neurons in ACJ mice co-expressed CGRP (Fig. 6I, J). Meanwhile, the percentage of FcεRIα^+^ sensory neurons was also increased among MrgprA3^+^ pruriceptors (from 13.38 to 28.07%). In addition, no obvious GS^+^ signal was detected within FcεRIα^+^ neurons innervating conjunctiva for both ACJ and control-treated mice (Fig. 6K, L). Moreover, we did not detect obvious immune cell infiltration within TG for ACJ mice compared with the control-treated mice (Additional file 1: Fig. S4). These findings suggest that the expression of FcεRIα in conjunctival sensory neurons is significantly upregulated under ACJ condition.

### Neuronal FcεRIα mediated allergic ocular itch in ACJ model

**Fig. 7:**
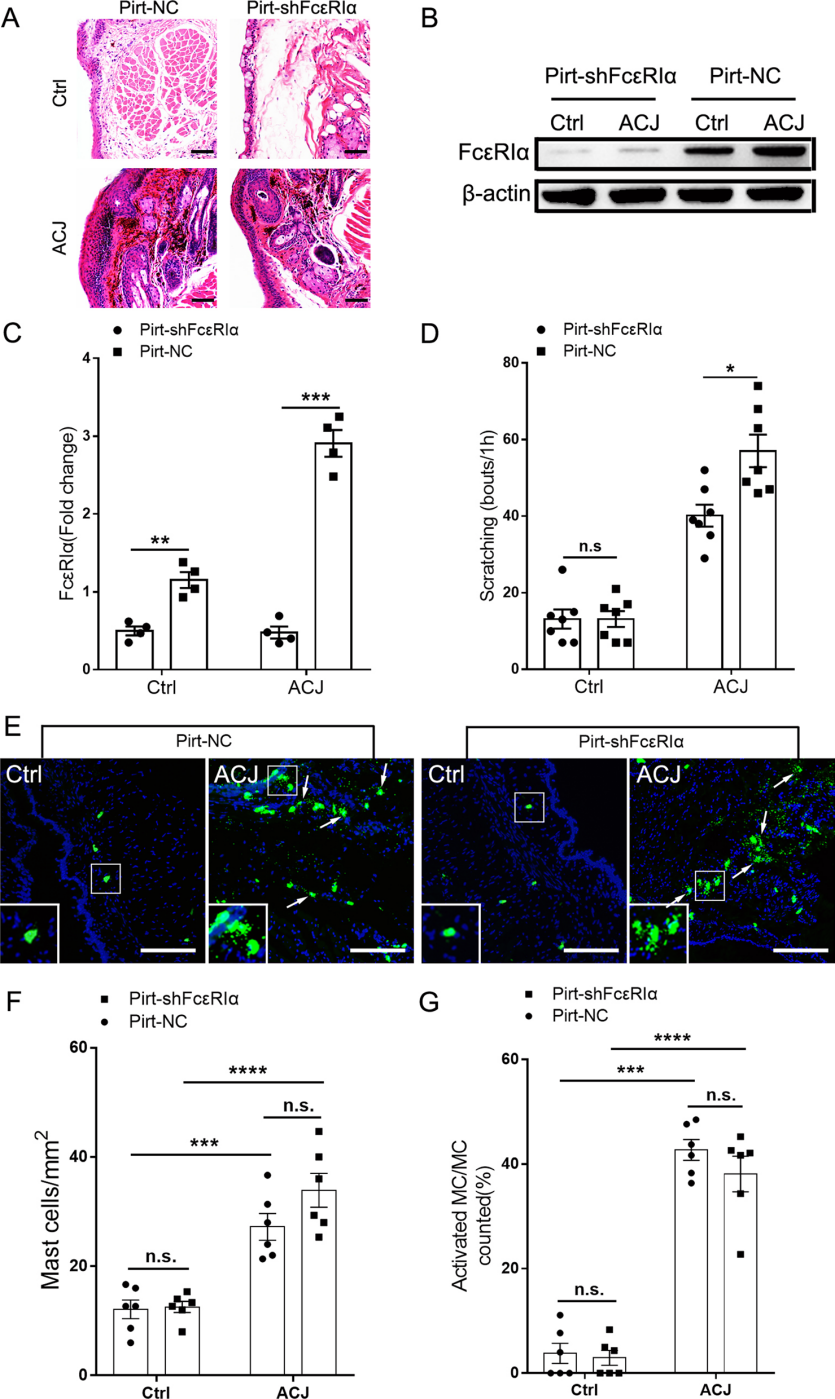
Neuronal FcεRIα mediates ocular itch in the ACJ murine model. **A** Representative H&E images of conjunctiva from mice infected by two types of viruses taken 12 h after ACJ induction. Scale bar: 100 μm. **B** Representative gel band of FcεRIα protein in TG from control or ACJ-treated mice after trigeminal injection of two types viruses. **C** Western blot revealed a significant decrease in the level of FcεRIα protein in Pirt-shFcεRIα group compared with Pirt-NC group for both control and ACJ-treated mice. *n* = 4 per group, **P < *0.01*, ***P < *0.001*, Pirt-shFcεRIα vs. Pirt-NC, Student’s *t*-test. **D** Eye-towards scratching behavior in ACJ model as challenged by OVA was significantly decreased in Pirt-shFcεRIα group compared with Pirt-NC group, while no difference was discovered in control-treated mice between two types viruses. *n* = 7 per group, *P < *0.05*, Pirt-shFcεRIα vs. Pirt-NC, Student’s *t*-test. **E** Representative images of conjunctiva from mice infected by two types viruses taken 12 h after ACJ induction and stained for FITC-avidin. The mast cells in allergic conjunctiva presented as irregular shape with degranulation. The embedded boxes showed the enlarged views. Scale bar: 100 μm. **F** Quantitative analysis of mast cells number after different treatments. *n* = 6 per group; ***P < *0.001*, ****P < *0.0001*, ACJ vs. Ctrl infected by the same virus, Student’s *t*-test. *n.s.* no significance, Pirt-shFcεRIα vs. Pirt-NC, Student’s *t*-test. **G** Proportions of activated mast cells (with degranulation) after different treatments. *n* = 6 per group; ***P < *0.001*, ****P < *0.0001*, ACJ vs. Ctrl infected by the same virus, Student’s *t*-test. *n.s.* no significance, Pirt-shFcεRIα vs. Pirt-NC, Student’s *t*-test. *Ctrl* control, *ACJ* allergic conjunctivitis

Given that FcεRIα was upregulated in CGRP^+^ nociceptors and MrgprA3^+^ pruriceptors in ACJ, and IgE-IC could evoke acute ocular itch through neuronal FcεRIα, we next asked whether neuronal FcεRIα was involved in ocular itch under allergic conjunctivitis condition. From 14th day after intra-TG virus injection (AAV9-pirt-shFcεRIα-EGFP and AAV9-pirt-NC-EGFP), we produced ACJ model. Following challenged by OVA, the ACJ pathology was induced in ACJ group (sensitized on day 0, and boosted on day 7 and day 14) mice, but not control-treated mice (challenged by OVA without any sensitization). However, no obvious difference was detected in H&E staining between the mice infected by AAV9-pirt-NC-EGFP and the ones infected by AAV9-pirt-shFcεRIα-EGFP, indicating that neuro-knockdown of FcεRIα did not affect the development of ACJ pathology (Fig. 7A). The Western blot results showed that trigeminal FcεRIα was significantly upregulated in ACJ model for mice infected by NC-loaded virus, whereas no alteration was detected for the shFcεRIα-loaded virus group. In addition, the trigeminal expression of FcεRIα was significantly downregulated in the AAV9-pirt-shFcεRIα-EGFP group compared with the AAV9-pirt-NC-EGFP group, both control- and ACJ-treated mice (Fig. 7B, C). As challenged by OVA, mice infected by the shFcεRIα-loaded virus showed significantly reduced ocular itch than those infected by the NC-loaded control virus (Fig. 7D). To determine the role of conjunctival inflammation in the reduced itch as neuro-knockdown of FcεRIα, IF was applied to assess the immune cell infiltration. Quantification showed a significant increase in Ly6C/G, IBA1, and CD3 in the conjunctiva following ACJ for mice infected by both two viruses. Notably, neuro-knockdown of FcεRIα in the AAV9-pirt-shFcεRIα-EGFP group showed no influence on the infiltration of neutrophils (Ly6C/G), macrophages (IBA1), and lymphocytes (CD3) following ACJ, compared with the AAV9-pirt-NC-EGFP group (Additional file 1: Fig. S5). In addition, we next examine the effects of neuron-specific knockdown FcεRIα on infiltration and activation of mast cells under ACJ conditions. The numbers of mast cells showed no difference in the AAV9-pirt-shFcεRIα-EGFP group compared with the AAV9-pirt-NC-EGFP group, for both control- and ACJ-treated mice. Moreover, the percentages of degranulated mast cells also showed no difference for the mice infected by AAV9-pirt-shFcεRIα-EGFP. Above all, these results suggested that neuronal FcεRIα directly mediated allergic ocular itch in ACJ model (Fig. 7E–G).

### IgE upregulated the neural expression of FcεRIα with elevated neural excitability in TG

**Fig. 8:**
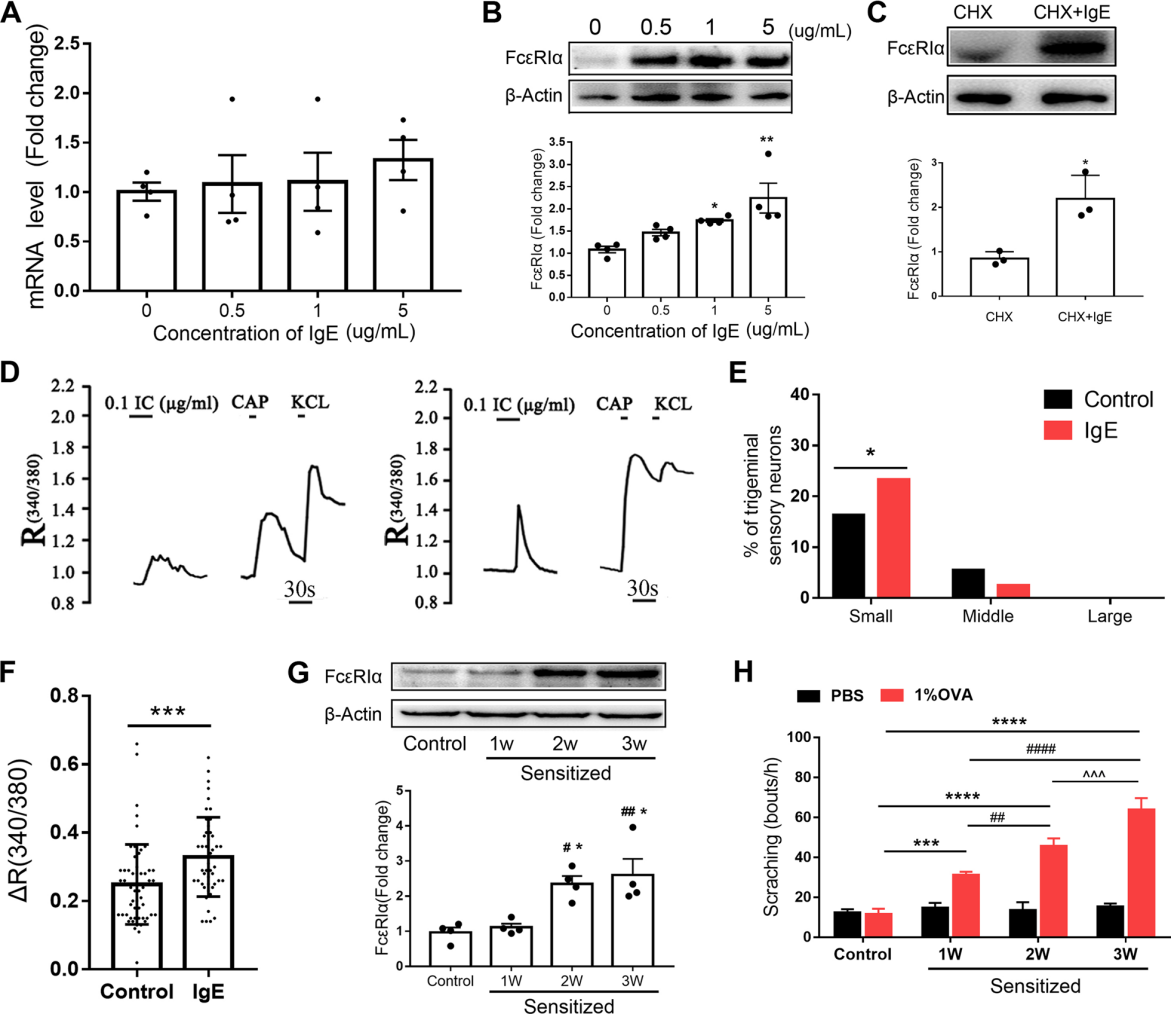
IgE upregulates neuronal FcεRIα and enhanced neuronal response to IgE-IC. **A** qRT-PCR revealed that FcεRIα mRNA was not changed by IgE. One-way ANOVA followed by Bonferroni’s post hoc test comparisons, *n* = 4. **B** Representative Western blotting gel bands of FcεRIα protein from dissociated TG neurons cultured with IgE of different concentrations. Western blotting revealed a significant increase in the protein levels of FcεRIα in dissociated TG neurons cultured with IgE. *P < *0.05*, **P < *0.01*, vs. Control, one-way ANOVA followed by Bonferroni’s post hoc test comparisons, *n* = 4. **C** IgE upregulated the FcεRIα protein in dissociated TG neurons as blocking protein synthesis by Cycloheximide (CHX). *P < *0.05*, CHX vs. CHX+IgE, Student’s *t*-test, *n* = 3. **D** TG neurons cultured with (right, IgE group) and without (left, Control group) IgE (5 μg/ml) were exposed to IgE-IC (IC; 0.1 μg/ml; 30 s), followed by capsaicin (CAP; 1 μM; 10 s) and KCl (50 mM; 10 s). Neuronal activation was analyzed by calcium imaging (representative trace). **E** Proportion of responsive small-diameter neurons as IgE-IC application was significantly increased in IgE group compared with Control group. *P < *0.05*, IgE vs. Control, *χ*^2^ test. **F** Mean magnitude of IgE-IC-induced *R* (340/380) growth was also elevated in IgE group. ***P < *0.001*, IgE vs. Control, Student’s *t*-test. **G** Protein levels of FcεRIα in TG from mice sensitized different times were determined by Western blotting. ^#^*P* < *0.05*, ^##^*P* < *0.01* vs. Control; *P < *0.05* vs. Sensitized 1w, one-way ANOVA followed by Bonferroni’s post hoc test comparisons. **H** Ocular scratching behavior as challenged by OVA (1% in PBS) or vehicle in mice sensitized different times. *n* = 6 per group, ***P < *0.001*, ****P < *0.0001* vs. Control; ^##^*P* < *0.0*1, ^####^*P* < *0.0001* vs. Sensitized 1w; ^^^*P* < *0.001* vs. Sensitized 2w; Student’s *t*-test

To explore the regulatory mechanism of neuronal FcεRIα, the mRNA and protein level of FcεRIα in dissociated TG neurons cultured by media containing different concentrations of isotype IgE were determined by qRT-PCR and Western blot. The FcεRIα protein increased significantly in dissociated TG neurons cultured with IgE without significant changes in the level of mRNA (Fig. 8A, B). Cycloheximide (10 μg/ml), a potent inhibitor of translation, failed to block the effect of IgE (5 μg/ml) in the upregulation of FcεRIα (Fig. 8C). To further examine the effects of IgE on the excitability of TG neurons, we conducted calcium imaging of the dissociated TG neurons (Fig. 8D). Calcium imaging showed that small-size neurons cultured with isotype IgE (5 μg/ml) responded to IgE-IC intensively. No difference was observed for middle-size and large-size neurons. The percentage of IgE-IC-responsive neurons and the magnitude of IgE-IC-evoked Ca^2+^ responses were significantly increased when IgE was added into the culture media (Fig. 8E, F). Calcium imaging was also conducted for dissociated neurons from *Mrgpra3*^*GFP-Cre*^ mice, which detected the responses of MrgprA3^+^ neurons to IgE-IC. For MrgprA3^+^ neurons, IgE (5 μg/ml) increased the percentage of IgE-IC-responsive neurons (from 25.22 to 39.02%) and the intensity (Δ*R* (340/380) from 22 to 30% baseline) of the Ca^2+^ responses (Additional file 1: Fig. S6). To in vivo observe the role of IgE for neuro-expression of FcεRIα, we designed an incremental sensitization workflow (Additional file 1: Fig. S7A). First, we detected elevated OVA-specific IgE in the serum of sensitized mice, suggesting that boost could sustain the level of IgE in the serum and extended sensitization increased the action time of IgE to sensory neurons (Additional file 1: Fig. S7B). We further found that levels of FcεRIα in TG neurons were significantly increased in mice sensitized 2 weeks (2w) and sensitized 3 weeks (3w) compared with mice of control-treated and sensitized 1 week (1w) (Fig. 8G). Immunofluorescent staining showed that the increased FcεRIα was mainly detected in small-sized TG neurons in the groups of sensitized 2w and sensitized 3w compared with control-treated group and sensitized 1w group (Additional file 1: Fig. S7C, D). To determine the effect of sensitized duration on the ocular pruritus degree, we dropped allergen (1% OVA in PBS) to the eyes of mice with increased times of sensitization. We found that sensitized mice presented scratching behavior as challenged by allergen, but not the unsensitized mice. Notably, incremental sensitization induced aggravating scratching behavior when treated with OVA (Fig. 8H). The above results indicate that IgE might upregulate neuronal FcεRIα and further intensify pruritus degree as antigen exposure.

## Discussion

From the conventional theory, FcεRI, a high-affinity activating receptor for IgE, is mainly expressed in allergy-related cells, such as mast cells and basophils. The cross-linking of FcεRI induced by the combination of IgE-IC with α subunit of FcεRI (FcεRIα) triggers degranulation of mast cells and release of inflammatory mediators, such as histamine, tryptase, platelet-activating factor, and prostaglandin E2 [34, 35]. Notably, we and others have previously reported that sensory neurons expressed FcεRI including all three subunits FcεRIα, FcεRIβ, and FcεRIγ. The present study focused on the IgE-binding subunit FcεRIα and further identified that FcεRIα was functionally and anatomically expressed in a subset of conjunctiva-innervating sensory neurons. We detected FcεRIα protein expression in conjunctival sensory neurons of NF200^+^ myelinated sensors and nociceptors, including both CGRP^+^ peptidergic and IB4^+^ nonpeptidergic neurons. Using calcium imaging, we also demonstrated that IgE-IC could directly activate trigeminal neurons in a manner dependent on FcεRIα. The distribution of other two subunits of FcεRI, FcεRIβ, and FcεRIγ, among conjunctiva-innervating sensor and their role in neuronal activation could be further identified to acquire a complete understanding of sensory FcεRI.

Another advance in this study is the revelation of a novel role for neuronal FcεRIα in ocular itch perception. MrgprA3, the pruriceptor of chloroquine, marked a subpopulation of conjunctiva-specific neurons that transmit acute and allergic-related ocular itch [29]. We detected FcεRIα expression in both the soma and the fiber of MrgprA3^+^ neurons, which could be directly activated by IgE-IC. Based on these discoveries, we investigated the effect of IgE-IC on the ocular sensation and the role of neuronal FcεRIα in it. First, conjunctival instillation of IgE-IC evoked eye-towards scratching behavior without obvious pain-related wiping, indicating that IgE-IC served as itch-specific regent for conjunctiva. Strikingly, this scratching behavior seemed to be dissociable from conjunctival inflammation, at least at an early stage, and the function of mast cells. Moreover, both global deletion and specifically Pirt-guided neuronal knockdown in trigeminal sensory neurons effectively alleviated the acute ocular itch induced by IgE-IC, suggesting that neuronal FcεRIα could independently mediate the acute ocular itch induced by IgE-IC.

Itch and pain are the main symptoms of various allergic conditions, which are considered as the consequence of intensive immune activation within the affected tissue [33, 36, 37]. However, current therapeutic strategies for these allergic-related nociception managements rely heavily on immune-suppressive drugs and often have limited efficacy or induced consequential complications. Recent studies demonstrated that FcγRI on joint-innervating sensor exhibited hypersensitivity and FcγRI-coupled signaling in peripheral nociceptor could mediate joint pain independently in allergic arthritis through conditional neuronal knockout animal model [38–40]. These studies indicated that peripheral sensitization of nociceptors mediated by the neuronal adaptive immune receptors as challenged by the immune complex might be a brand-new mechanism for allergic-related nociception.

Based on the clinical evidence that IgE was increased in the serum of allergic conjunctivitis patients and the above laboratory observation, the role of neuronal FcεRI in ocular itch under allergic conjunctivitis was further explored through ACJ murine model. The conjunctiva-innervating sensors increasingly expressed FcεRIα which co-expressed CGRP at a greater proportion in ACJ model. Previous studies reported that CGRP^+^ sensory neurons encoded itch and presented anatomical specificity in the eye, more detected in the conjunctiva-innervating neurons than in the cornea-innervating neurons [29, 41]. Moreover, a higher percentage of trigeminal MrgprA3^+^ pruriceptors expressed FcεRIα in ACJ model, suggesting the upregulation of FcεRIα in pruriceptors under ACJ condition. Our previous study showed that pharmacological antagonism to FcεRIα could alleviate ocular itch in ACJ murine model, while this outcome might result from disturbance to neuronal FcεRIα and/or FcεRIα in mast cells [25]. In the present study, we applied neuronal-selective knockdown of FcεRIα which significantly alleviated ocular itch as challenged by antigens, while the immune cell infiltration and mast cell activation were not altered. These findings provide promising and unique neuronal targets for new anti-ocular itch therapeutic strategies in ACJ.

More recently, transient receptor potential canonical (TRPC)3/6/7 were identified as the key downstream effective channels in FcεRI signaling in mast cells [42]. A previous study also demonstrated that neuronal FcγRI was functionally coupled to TRPC3 to regulate the excitability of DRG neurons [43]. In addition, histamine receptor 1 (H1R) coupled TRPV1 to mediate histamine-dependent ocular itch, while platelet activating factor receptor (PAFR) engaged TRPA1 to consist the histamine-independent ocular itch pathway [30]. The intracellular signaling and effective ion channel of FcεRI in sensory neurons should be further explored. To develop new therapy for itch symptom of ACJ patients, it is important to translate our discoveries from mice to humans [29]. Although the FcεRIα was detected in the human trigeminal ganglion, the functional identification of neuronal FcεRIα in humans and its changes under allergic conditions could be further studied. The application of humanized mice containing the human FcεRI receptor would also enhance the clinical significance of this study.

The regulatory mechanism of FcεRIα has been researched in immune cells which was involved in multiple factors and mechanisms. IgE was found to upregulate FcεRIα even under the condition of blocking synthesis of protein in basophils, which might result from decreased internalization and degradation of membranal FcεRIα [44–48]. However, FcεRIα localized in endolysosomes of DCs and was responsible for the clearance of IgE by internalization [49]. Cytokines such as IL-3 and TGF-β also regulated the expression of FcεRIα by affecting the transcription in immune cells [50, 51]. In our previous study, elevated mRNA and protein of FcεRIα in TG was also observed in allergic conjunctivitis model [25]. Given the complex changes in pathology and molecule induced by challenge, we used the allergic conjunctivitis model receiving incremental sensitization without challenge and dissociated neurons cultured with IgE to specifically research the effect of IgE in neuronal FcεRIα expression. The results demonstrated that IgE could upregulate neuronal FcεRIα without affecting transcription and translation and enhance the neuronal reaction to IgE-immune complex. This suggested that sensory neuron shared the similar regulatory mechanism of FcεRIα in basophils. The combination of IgE with membranal FcεRIα in sensory neuron might decrease its internalization and degradation. However, these results were not direct evidence for the above mechanism as the increase of membranal FcεRIα by IgE was still not certified. This issue could be further investigated by specifically labeling membranal FcεRIα in following studies. Overall, IgE not only sensitized the immune cells to be more sensitive to allergen, as well as the peripheral sensory neurons. These results illuminated the regulation mechanism of neuronal FcεRIα by IgE and may suggest therapeutic strategies targeting peripheral sensory neurons for itch.

Our findings revealed a novel mechanism of allergic ocular itch via direct activation of FcεRI in conjunctiva-innervating sensors by IgE-IC. The FcεRI receptor located in sensory neurons and immune cells may work together to contribute to ocular itch accompanying ACJ in mice and humans by directly activating itchy sensors and/or indirectly stimulating immune cells to induce the release of inflammatory mediators that target primary sensory neurons.

## Conclusions

This study demonstrates that FcεRIα in sensory neurons innervating conjunctiva directly mediates IgE-IC induced acute ocular itch. Furthermore, ACJ upregulates FcεRIα in sensory neurons innervating conjunctiva, which mediates ACJ-related ocular itch as antigen exposure. In addition, IgE upregulates FcεRIα protein in sensory neurons and enhances neuronal responses to IgE-IC. Incremental sensitization would intensity ocular itch as antigen exposure, which might result from upregulated FcεRIα protein in sensory neurons. These findings reveal a novel neuroimmune axis in allergic itch condition, which highlights the immunosensory capabilities of the itchy neurons.

## Supplementary information

**Additional file 1: Figure S1.** Expression of FcεRIα in MrgprA3^+^ pruriceptors. **A**, **B** Expression of FcεRIα in trigeminal ganglion (**A**) and spleen (**B**) of *FcεRIα*^*−/−*^ mice. Scale bar: 50 μm. **C** Immunostaining by isotype IgG in the TG of WT mice. Scale bar: 50 μm. **D** Detection of FcεRIα by immunostaining in the TG of *Mrgpra3*^*GFP-cre*^ mice. Scale bar: 100 μm. **E** Proportion of FcεRIα^+^ neurons among Mrgpra3^+^ pruriceptors. **F**–**H** Detection of FcεRIα by immunostaining in the conjunctiva of *Mrgpra3*^*GFP-Cre*^; *ROSA26*^*tdTomato*^ mice. Scale bar: 20 μm. **Figure S2.** IgE-IC directly activates MrgprA3^+^ pruriceptors in vitro. **A** Identification of MrgprA3^+^ trigeminal neuron by fluorescent view. **B**–**D** Representative fluorescent view and Fura-2 ratiometric imaging of dissociated MrgprA3^+^ neuron (red arrow) and MrgprA3^−^ neuron (white arrow). Scale bar = 50 μm. **Figure S3.** IgE-IC does not cause immune cell infiltration in mouse TG. **A** Mice were ocular instilled with IgE-IC (1, 10, 50 μg/ml; 5 μl), monomeric IgE (50 μg/ml; 5 μl), OVA (100 mg/ml, 5 μl) or vehicle (PBS; 5 μl), and eye-towards wiping bouts were counted over 1–12 h. The baseline was identified as recorded without any instillation. *n* = 8–10 mice per group; 2-way ANOVA for repeated measures followed by Bonferroni’s post hoc test. **B**–**D** Representative images of trigeminal ganglions which were taken 1 h after ocular instillation with either PBS, monomeric IgE, or IgE-IC and stained for Ly6C/G, IBA1, and CD3. Scale bar: 100 μm. **E** Quantification showed no significant differences in fluorescence intensity of markers among treatment groups. *n* = 4 per group; one-way ANOVA followed by Bonferroni’s post hoc test comparisons. **F** Representative images of trigeminal ganglions which were taken 1 h after ocular instillation with either PBS, monomeric IgE, or IgE-IC and stained for FITC-avidin. Scale bar: 100 μm. **G** Quantitative analysis of mast cells number in the TG after different treatments. *n* = 4 per group; one-way ANOVA followed by Bonferroni’s post hoc test comparisons. **H** Proportions of activated mast cells (with degranulation) in TG after different treatments. *n* = 4 per group; one-way ANOVA followed by Bonferroni’s post hoc test comparisons. **Figure S4.** ACJ does not cause immune cell infiltration in mouse TG. **A** Representative images of mouse TG sections stained for Ly6C/G (green) and PGP9.5 (red) 12 h after ACJ induction or control-treated mice. **B** Quantification showed no significant difference in fluorescence intensity of Ly6C/G between ACJ and control-treated mice. *n* = 4 mice per group; *n.s.* no significance, Student’s *t*-test. **C** Representative images of mouse TG sections stained for IBA1 (green) and PGP9.5 (red) 12 h after ACJ induction or control-treated mice. **D** Quantification showed no significant difference in fluorescence intensity of IBA1 between ACJ and control-treated mice. *n* = 4 mice per group; *n.s.* no significance, Student’s *t*-test. **E** Representative images of mouse TG sections stained for CD3 (green) and PGP9.5 (red) 12 h after ACJ induction or control-treated mice. **F** Quantification showed no significant difference in fluorescence intensity of CD3 between ACJ and control-treated mice. *n* = 4 mice per group; *n.s.* no significance, Student’s *t*-test. **G** Representative images of mouse TG sections stained for FITC-avidin (green) and PGP9.5 (red) 12 h after ACJ induction or control-treated mice. **H** Quantitative analysis of mast cells number in TG from ACJ mice or control-treated mice. *n* = 4 per group. *n.s.* no significance, Student’s *t*-test. **I** Proportions of activated mast cells (with degranulation) in TG from ACJ mice or control-treated mice. *n* = 4 per group. *n.s.* no significance, Student’s *t*-test. **Figure S5.** Contribution of neuronal FcεRIα in conjunctival inflammation at 12th hour following ACJ. Representative conjunctiva sections from mice infected by AAV9-Pirt-NC-EGFP or AAV9-Pirt-shFcεRIα-EGFP after OVA challenge and stained for Ly6C/G (**A**), IBA1 (**C**), and CD3 (**E**). Scale bar, 100 µm. The embedded boxes showed the enlarged views. **B** Quantification for fluorescence intensity of Ly6C/G showed no significant differences as comparing Control- or ACJ-treated mice between two viruses’ groups (Left). Quantitative analysis of Ly6C/G+ cell numbers showed no significant differences as comparing Control- or ACJ-treated mice between two viruses’ groups (Right). *n* = 4 per group; Student’s *t*-test. **D** Quantification for fluorescence intensity of IBA1 showed no significant differences as comparing Control- or ACJ-treated mice between two viruses’ groups (Left). Quantitative analysis of IBA1+ cell numbers showed no significant differences as comparing Control- or ACJ-treated mice between two viruses’ groups (Right). *n* = 4 per group; Student’s *t*-test. **F** Quantification for fluorescence intensity of CD3 showed no significant differences as comparing Control- or ACJ-treated mice between two viruses’ groups (Left). Quantitative analysis of CD3+ cell numbers showed no significant differences as comparing Control- or ACJ-treated mice between two viruses’ groups (Right). *n* = 4 per group; Student’s *t*-test. *n.s.* no significance, AAV9-Pirt-NC-EGFP vs. AAV9-Pirt-shFcεRIα-EGFP. **Figure S6.** IgE enhances Ca^2+^ response of MrgprA3^+^ trigeminal neurons to IgE-IC. **A** The percentage of IgE-IC (0.1 μg/ml) responsive MrgprA3^+^ neurons was significantly increased as cultured with IgE (5 μg/ml). *P < *0.05*, IgE vs. Control, *χ*^2^ test. **B** Magnitude of IgE-IC-evoked Ca^2+^ responses was significantly increased for MrgprA3^+^ neurons cultured with IgE (5 μg/ml) in *MrgPra3*^*GFP-Cre*^ mice. *P < *0.05*, IgE vs. Control, Student’s *t*-test. **Figure S7.** Incremental sensitization gradually upregulates trigeminal FcεRIα in small-diameter neurons. **A** Schematic diagram of experimental design for incremental sensitization model. **B** Determination of anti-OVA IgE in the serum of each group. *P < *0.05*, vs. Control, one-way ANOVA followed by Bonferroni’s post hoc test comparisons. **C** Typical images for the immunofluorescence staining of FcεRIα (green) and PGP9.5 (red) in the Control, Sensitized 1w, Sensitized 2w and Sensitized 3w mice. Scale bar: 25 μm. **D** Percentage of FcεRIα-immunopositive TG neurons from mice receiving sensitization of different times (*n* > 100 neurons total for each group). *P < *0.05*, vs. Control; ^#^*P* < *0.05*, vs. sensitized 1w, *χ*^2^ test.

**Additional file 2: Table S1.** List of antibodies. List of antibodies for IF.

## Data Availability

There are no data, software, databases, and application/tools available apart from those reported in the present study. All data are provided in the manuscript and Additional files 1, 2.

## Abbreviations

AAV: Adeno-associated virus
ACJ: Allergic conjunctivitis
CGRP: Calcitonin gene-related peptide
DRG: Dorsal root ganglion
FcγRI: Fc-gamma-receptor I
FcεRI: Fc-epsilon receptor I
His: Histamine
IB4: Isolectin B4
IC: Immune complex
IgE: Immunoglobulin E
IgG: Immunoglobulin G
ITAMs: Immunoreceptor tyrosine-based activation motifs
OVA: Ovalbumin
PAFR: Platelet activating factor receptor
TG: Trigeminal ganglion

## References

[1] Rosario N, Bielory L (2011). Epidemiology of allergic conjunctivitis. Curr Opin Allergy Clin Immunol.

[2] Ono SJ, Abelson MB (2005). Allergic conjunctivitis: update on pathophysiology and prospects for future treatment. J Allergy Clin Immunol.

[3] Fukuda K, Ohbayashi M, Morohoshi K, Zhang L, Liu F-T, Ono SJ (2009). Critical role of IgE-dependent mast cell activation in a murine model of allergic conjunctivitis. J Allergy Clin Immunol.

[4] Dong X, Dong X (2018). Peripheral and central mechanisms of itch. Neuron.

[5] Liu Q, Dong X (2015). The role of the Mrgpr receptor family in itch. Handb Exp Pharmacol.

[6] LaMotte RH, Dong X, Ringkamp M (2014). Sensory neurons and circuits mediating itch. Nat Rev Neurosci.

[7] Zhao J, Munanairi A, Liu XY, Zhang J, Hu L, Hu M, Bu D, Liu L, Xie Z, Kim BS (2020). PAR2 mediates itch via TRPV3 signaling in keratinocytes. J Invest Dermatol.

[8] Simons FE, Simons KJ (2011). Histamine and H1-antihistamines: celebrating a century of progress. J Allergy Clin Immunol.

[9] Bielory L (2008). Ocular allergy treatment. Immunol Allergy Clin North Am.

[10] Amirian ES, Marquez-Do D, Bondy ML, Scheurer ME (2013). Antihistamine use and immunoglobulin E levels in glioma risk and prognosis. Cancer Epidemiol.

[11] Yamana Y, Fukuda K, Ko R, Uchio E (2019). Local allergic conjunctivitis: a phenotype of allergic conjunctivitis. Int Ophthalmol.

[12] Mimura T, Yamagami S, Kamei Y, Goto M, Matsubara M (2013). Specific IgE in tear fluid and features of allergic conjunctivitis. Curr Eye Res.

[13] Jensen RK, Jabs F, Miehe M, Mølgaard B, Pfützner W, Möbs C, Spillner E, Andersen GR (2020). Structure of intact IgE and the mechanism of ligelizumab revealed by electron microscopy. Allergy.

[14] Corry DB, Kheradmand F (1999). Induction and regulation of the IgE response. Nature.

[15] Turner H, Kinet JP (1999). Signalling through the high-affinity IgE receptor Fc epsilonRI. Nature.

[16] Siraganian RP (2003). Mast cell signal transduction from the high-affinity IgE receptor. Curr Opin Immunol.

[17] Gounni AS, Lamkhioued B, Delaporte E, Dubost A, Kinet JP, Capron A, Capron M (1994). The high-affinity IgE receptor on eosinophils: from allergy to parasites or from parasites to allergy?. J Allergy Clin Immunol.

[18] Alber G, Kent UM, Metzger H (1992). Functional comparison of Fc epsilon RI, Fc gamma RII, and Fc gamma RIII in mast cells. J Immunol.

[19] Garman SC, Kinet JP, Jardetzky TS (1998). Crystal structure of the human high-affinity IgE receptor. Cell.

[20] Garman SC, Wurzburg BA, Tarchevskaya SS, Kinet JP, Jardetzky TS (2000). Structure of the Fc fragment of human IgE bound to its high-affinity receptor Fc epsilonRI alpha. Nature.

[21] Gevaert P, Omachi TA, Corren J, Mullol J, Han J, Lee SE, Kaufman D, Ligueros-Saylan M, Howard M, Zhu R (2020). Efficacy and safety of omalizumab in nasal polyposis: 2 randomized phase 3 trials. J Allergy Clin Immunol.

[22] Heffler E, Picardi G, Liuzzo MT, Pistorio MP, Crimi N (2016). Omalizumab treatment of vernal keratoconjunctivitis. JAMA Ophthalmol.

[23] van der Kleij H, Charles N, Karimi K, Mao YK, Foster J, Janssen L, Chang Yang P, Kunze W, Rivera J, Bienenstock J (2010). Evidence for neuronal expression of functional Fc (epsilon and gamma) receptors. J Allergy Clin Immunol.

[24] Andoh T, Kuraishi Y (2004). Expression of Fc epsilon receptor I on primary sensory neurons in mice. NeuroReport.

[25] Liu F, Xu L, Chen N, Zhou M, Li C, Yang Q, Xie Y, Huang Y, Ma C (2017). Neuronal Fc-epsilon receptor I contributes to antigen-evoked pruritus in a murine model of ocular allergy. Brain Behav Immun.

[26] Liu Q, Tang Z, Surdenikova L, Kim S, Patel KN, Kim A, Ru F, Guan Y, Weng HJ, Geng Y (2009). Sensory neuron-specific GPCR Mrgprs are itch receptors mediating chloroquine-induced pruritus. Cell.

[27] Han L, Ma C, Liu Q, Weng HJ, Cui Y, Tang Z, Kim Y, Nie H, Qu L, Patel KN (2013). A subpopulation of nociceptors specifically linked to itch. Nat Neurosci.

[28] Zhao H, Yang H, Geng C, Chen Y, Pang J, Shu T, Zhao M, Tang Y, Li Z, Li B (2021). Role of IgE-FcεR1 in pathological cardiac remodeling and dysfunction. Circulation.

[29] Huang CC, Yang W, Guo C, Jiang H, Li F, Xiao M, Davidson S, Yu G, Duan B, Huang T (2018). Anatomical and functional dichotomy of ocular itch and pain. Nat Med.

[30] Huang CC, Kim YS, Olson WP, Li F, Guo C, Luo W, Huang AJW, Liu Q (2016). A histamine-independent itch pathway is required for allergic ocular itch. J Allergy Clin Immunol.

[31] Zhang Q, Cao DL, Zhang ZJ, Jiang BC, Gao YJ (2016). Chemokine CXCL13 mediates orofacial neuropathic pain via CXCR5/ERK pathway in the trigeminal ganglion of mice. J Neuroinflamm.

[32] Su W, Yu J, Zhang X, Ma L, Huang Y (2021). Proteome profile of trigeminal ganglion in murine model of allergic contact dermatitis: complement 3 pathway contributes to itch and pain sensation. Neurotox Res.

[33] Jiang H, Cui H, Wang T, Shimada SG, Sun R, Tan Z, Ma C, LaMotte RH (2019). CCL2/CCR2 signaling elicits itch- and pain-like behavior in a murine model of allergic contact dermatitis. Brain Behav Immun.

[34] Kubo M (2018). Mast cells and basophils in allergic inflammation. Curr Opin Immunol.

[35] Boyce JA (2003). Mast cells: beyond IgE. J Allergy Clin Immunol.

[36] Pall PS, Hurwitz OE, King BA, LaMotte RH (2015). Psychophysical measurements of itch and nociceptive sensations in an experimental model of allergic contact dermatitis. J Pain.

[37] Leuchtweis J, Segond von Banchet G, Eitner A, Ebbinghaus M, Schaible HG (2020). Pain-related behaviors associated with persistence of mechanical hyperalgesia after antigen-induced arthritis in rats. Pain.

[38] Jiang H, Shen X, Chen Z, Liu F, Wang T, Xie Y, Ma C (2017). Nociceptive neuronal Fc-gamma receptor I is involved in IgG immune complex induced pain in the rat. Brain Behav Immun.

[39] Liu F, Shen X, Su S, Cui H, Fang Y, Wang T, Zhang L, Huang Y, Ma C (2020). Fcγ receptor I-coupled signaling in peripheral nociceptors mediates joint pain in a rat model of rheumatoid arthritis. Arthritis Rheumatol.

[40] Wang L, Jiang X, Zheng Q, Jeon SM, Chen T, Liu Y, Kulaga H, Reed R, Dong X, Caterina MJ, Qu L (2019). Neuronal FcγRI mediates acute and chronic joint pain. J Clin Invest.

[41] McCoy ES, Taylor-Blake B, Street SE, Pribisko AL, Zheng J, Zylka MJ (2013). Peptidergic CGRPα primary sensory neurons encode heat and itch and tonically suppress sensitivity to cold. Neuron.

[42] Sanchez-Miranda E, Ibarra-Sanchez A, Gonzalez-Espinosa C (2010). Fyn kinase controls FcepsilonRI receptor-operated calcium entry necessary for full degranulation in mast cells. Biochem Biophys Res Commun.

[43] Qu L, Li Y, Pan X, Zhang P, LaMotte RH, Ma C (2012). Transient receptor potential canonical 3 (TRPC3) is required for IgG immune complex-induced excitation of the rat dorsal root ganglion neurons. J Neurosci.

[44] Furuichi K, Rivera J, Isersky C (1985). The receptor for immunoglobulin E on rat basophilic leukemia cells: effect of ligand binding on receptor expression. Proc Natl Acad Sci USA.

[45] MacGlashan D (2008). IgE receptor and signal transduction in mast cells and basophils. Curr Opin Immunol.

[46] MacGlashan D, Xia HZ, Schwartz LB, Gong J (2001). IgE-regulated loss, not IgE-regulated synthesis, controls expression of FcepsilonRI in human basophils. J Leukoc Biol.

[47] Conner ER, Saini SS (2005). The immunoglobulin E receptor: expression and regulation. Curr Allergy Asthma Rep.

[48] Kubo S, Matsuoka K, Taya C, Kitamura F, Takai T, Yonekawa H, Karasuyama H (2001). Drastic up-regulation of Fcepsilonri on mast cells is induced by IgE binding through stabilization and accumulation of Fcepsilonri on the cell surface. J Immunol.

[49] Greer AM, Wu N, Putnam AL, Woodruff PG, Wolters P, Kinet JP, Shin JS (2014). Serum IgE clearance is facilitated by human FcεRI internalization. J Clin Invest.

[50] Zellweger F, Buschor P, Hobi G, Brigger D, Dahinden CA, Villiger PM, Eggel A (2018). IL-3 but not monomeric IgE regulates FcεRI levels and cell survival in primary human basophils. Cell Death Dis.

[51] Yamazaki S, Nakano N, Honjo A, Hara M, Maeda K, Nishiyama C, Kitaura J, Ohtsuka Y, Okumura K, Ogawa H, Shimizu T (2015). The transcription factor Ehf is involved in TGF-β-induced suppression of FcεRI and c-Kit expression and FcεRI-mediated activation in mast cells. J Immunol.

