# Supplementary figures and images for: Neuronal FcεRIα directly mediates ocular itch via IgE-immune complex in a mouse model of allergic conjunctivitis
Source: J Neuroinflammation. 2022 Feb 23;19:55. doi: 10.1186/s12974-022-02417-x (PMC8867756; doi:10.1186/s12974-022-02417-x)

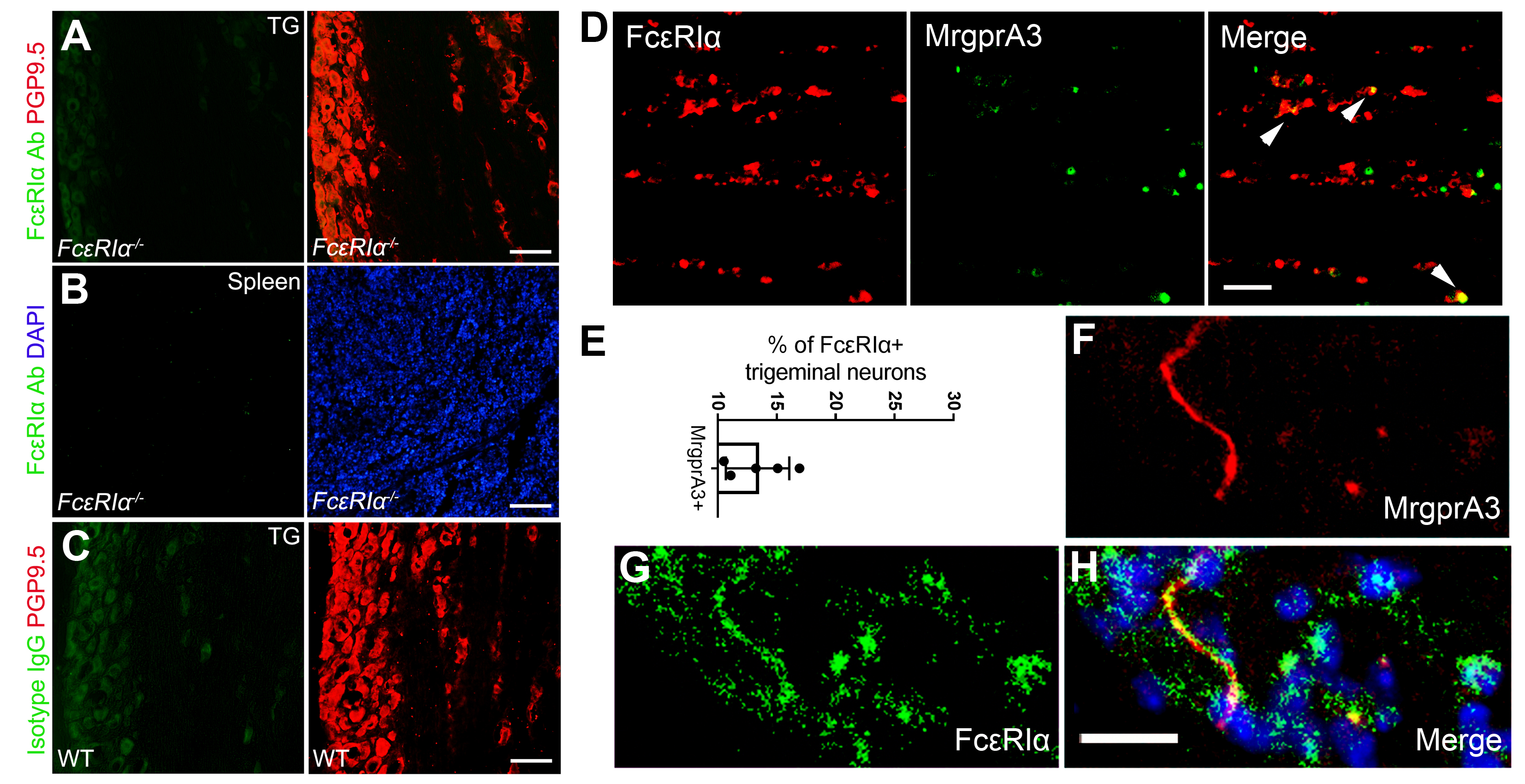

Supplement: Supplementary file 1 — Additional file 1: Figure S1. Expression of FcεRIα in MrgprA3+ pruriceptors. A, B Expression of FcεRIα in trigeminal ganglion (A) and spleen (B) of FcεRIα−/− mice. Scale bar: 50 μm. C Immunostaining by isotype IgG in the TG of WT mice. Scale bar: 50 μm. D Detection of FcεRIα by immunostaining in the TG of Mrgpra3GFP-cre mice. Scale bar: 100 μm. E Proportion of FcεRIα+ neurons among Mrgpra3+ pruriceptors. F–H Detection of FcεRIα by immunostaining in the conjunctiva of Mrgpra3GFP-Cre; ROSA26tdTomato mice. Scale bar: 20 μm. Figure S2. IgE-IC directly activates MrgprA3+ pruriceptors in vitro. A Identification of MrgprA3+ trigeminal neuron by fluorescent view. B–D Representative fluorescent view and Fura-2 ratiometric imaging of dissociated MrgprA3+ neuron (red arrow) and MrgprA3− neuron (white arrow). Scale bar = 50 μm. Figure S3. IgE-IC does not cause immune cell infiltration in mouse TG. A Mice were ocular instilled with IgE-IC (1, 10, 50 μg/ml; 5 μl), monomeric IgE (50 μg/ml; 5 μl), OVA (100 mg/ml, 5 μl) or vehicle (PBS; 5 μl), and eye-towards wiping bouts were counted over 1–12 h. The baseline was identified as recorded without any instillation. n = 8–10 mice per group; 2-way ANOVA for repeated measures followed by Bonferroni’s post hoc test. B–D Representative images of trigeminal ganglions which were taken 1 h after ocular instillation with either PBS, monomeric IgE, or IgE-IC and stained for Ly6C/G, IBA1, and CD3. Scale bar: 100 μm. E Quantification showed no significant differences in fluorescence intensity of markers among treatment groups. n = 4 per group; one-way ANOVA followed by Bonferroni’s post hoc test comparisons. F Representative images of trigeminal ganglions which were taken 1 h after ocular instillation with either PBS, monomeric IgE, or IgE-IC and stained for FITC-avidin. Scale bar: 100 μm. G Quantitative analysis of mast cells number in the TG after different treatments. n = 4 per group; one-way ANOVA followed by Bonferroni’s post hoc test compari [file 12974_2022_2417_MOESM1_ESM.zip › 12974_2022_2417_MOESM1_ESM/Figure-S1.jpg]

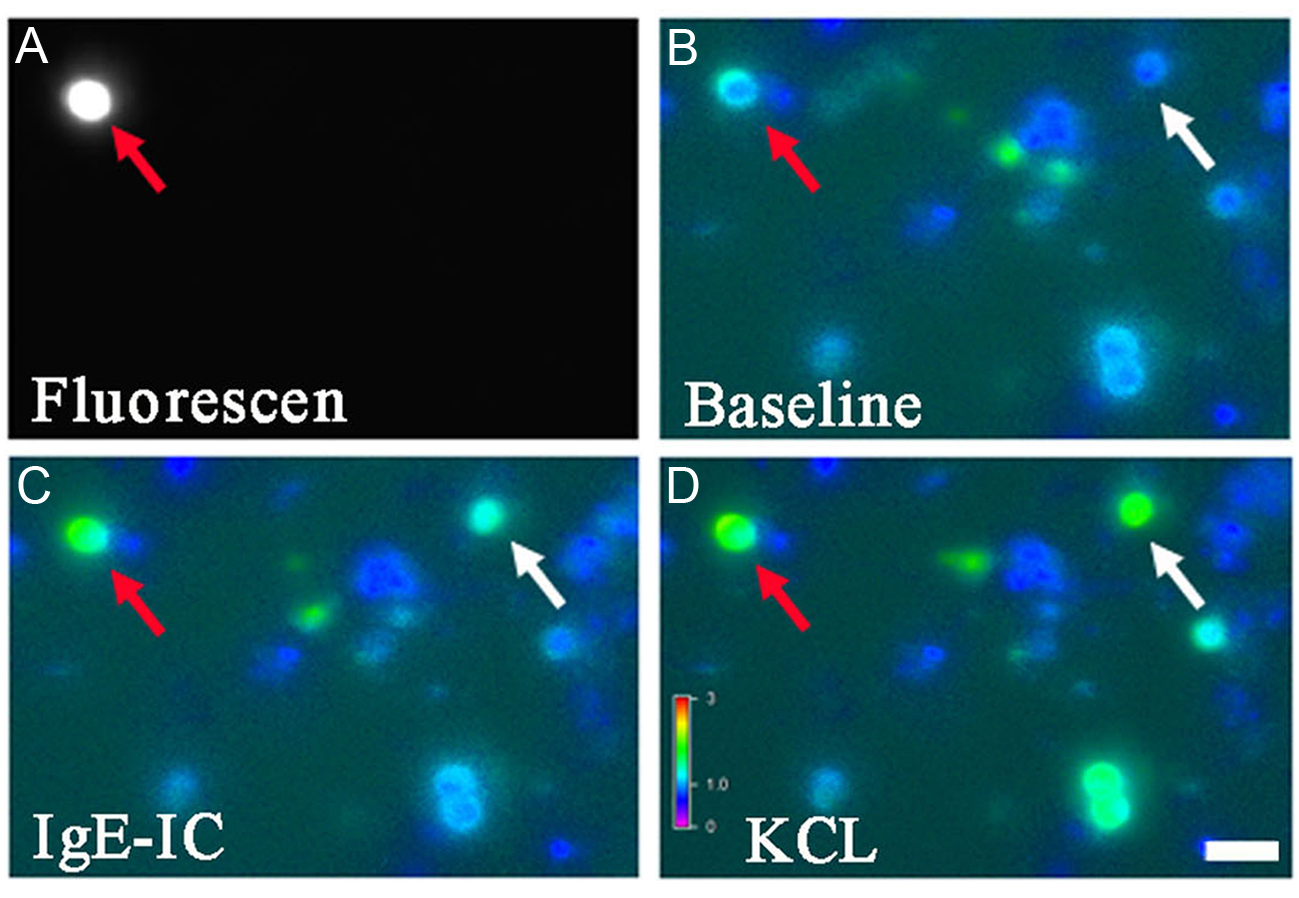

Supplement: Supplementary file 1 — Additional file 1: Figure S1. Expression of FcεRIα in MrgprA3+ pruriceptors. A, B Expression of FcεRIα in trigeminal ganglion (A) and spleen (B) of FcεRIα−/− mice. Scale bar: 50 μm. C Immunostaining by isotype IgG in the TG of WT mice. Scale bar: 50 μm. D Detection of FcεRIα by immunostaining in the TG of Mrgpra3GFP-cre mice. Scale bar: 100 μm. E Proportion of FcεRIα+ neurons among Mrgpra3+ pruriceptors. F–H Detection of FcεRIα by immunostaining in the conjunctiva of Mrgpra3GFP-Cre; ROSA26tdTomato mice. Scale bar: 20 μm. Figure S2. IgE-IC directly activates MrgprA3+ pruriceptors in vitro. A Identification of MrgprA3+ trigeminal neuron by fluorescent view. B–D Representative fluorescent view and Fura-2 ratiometric imaging of dissociated MrgprA3+ neuron (red arrow) and MrgprA3− neuron (white arrow). Scale bar = 50 μm. Figure S3. IgE-IC does not cause immune cell infiltration in mouse TG. A Mice were ocular instilled with IgE-IC (1, 10, 50 μg/ml; 5 μl), monomeric IgE (50 μg/ml; 5 μl), OVA (100 mg/ml, 5 μl) or vehicle (PBS; 5 μl), and eye-towards wiping bouts were counted over 1–12 h. The baseline was identified as recorded without any instillation. n = 8–10 mice per group; 2-way ANOVA for repeated measures followed by Bonferroni’s post hoc test. B–D Representative images of trigeminal ganglions which were taken 1 h after ocular instillation with either PBS, monomeric IgE, or IgE-IC and stained for Ly6C/G, IBA1, and CD3. Scale bar: 100 μm. E Quantification showed no significant differences in fluorescence intensity of markers among treatment groups. n = 4 per group; one-way ANOVA followed by Bonferroni’s post hoc test comparisons. F Representative images of trigeminal ganglions which were taken 1 h after ocular instillation with either PBS, monomeric IgE, or IgE-IC and stained for FITC-avidin. Scale bar: 100 μm. G Quantitative analysis of mast cells number in the TG after different treatments. n = 4 per group; one-way ANOVA followed by Bonferroni’s post hoc test compari [file 12974_2022_2417_MOESM1_ESM.zip › 12974_2022_2417_MOESM1_ESM/Figure-S2.jpg]

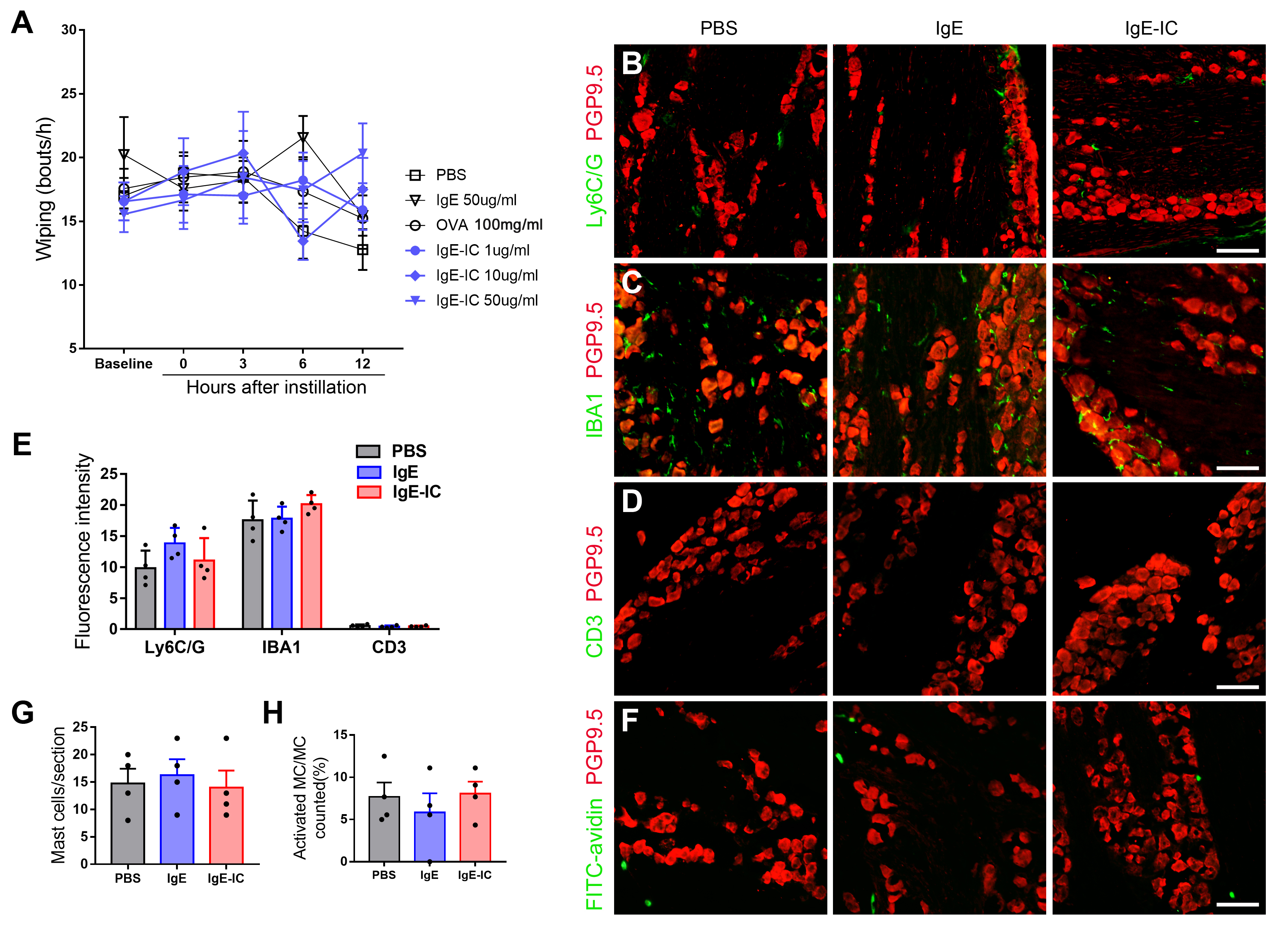

Supplement: Supplementary file 1 — Additional file 1: Figure S1. Expression of FcεRIα in MrgprA3+ pruriceptors. A, B Expression of FcεRIα in trigeminal ganglion (A) and spleen (B) of FcεRIα−/− mice. Scale bar: 50 μm. C Immunostaining by isotype IgG in the TG of WT mice. Scale bar: 50 μm. D Detection of FcεRIα by immunostaining in the TG of Mrgpra3GFP-cre mice. Scale bar: 100 μm. E Proportion of FcεRIα+ neurons among Mrgpra3+ pruriceptors. F–H Detection of FcεRIα by immunostaining in the conjunctiva of Mrgpra3GFP-Cre; ROSA26tdTomato mice. Scale bar: 20 μm. Figure S2. IgE-IC directly activates MrgprA3+ pruriceptors in vitro. A Identification of MrgprA3+ trigeminal neuron by fluorescent view. B–D Representative fluorescent view and Fura-2 ratiometric imaging of dissociated MrgprA3+ neuron (red arrow) and MrgprA3− neuron (white arrow). Scale bar = 50 μm. Figure S3. IgE-IC does not cause immune cell infiltration in mouse TG. A Mice were ocular instilled with IgE-IC (1, 10, 50 μg/ml; 5 μl), monomeric IgE (50 μg/ml; 5 μl), OVA (100 mg/ml, 5 μl) or vehicle (PBS; 5 μl), and eye-towards wiping bouts were counted over 1–12 h. The baseline was identified as recorded without any instillation. n = 8–10 mice per group; 2-way ANOVA for repeated measures followed by Bonferroni’s post hoc test. B–D Representative images of trigeminal ganglions which were taken 1 h after ocular instillation with either PBS, monomeric IgE, or IgE-IC and stained for Ly6C/G, IBA1, and CD3. Scale bar: 100 μm. E Quantification showed no significant differences in fluorescence intensity of markers among treatment groups. n = 4 per group; one-way ANOVA followed by Bonferroni’s post hoc test comparisons. F Representative images of trigeminal ganglions which were taken 1 h after ocular instillation with either PBS, monomeric IgE, or IgE-IC and stained for FITC-avidin. Scale bar: 100 μm. G Quantitative analysis of mast cells number in the TG after different treatments. n = 4 per group; one-way ANOVA followed by Bonferroni’s post hoc test compari [file 12974_2022_2417_MOESM1_ESM.zip › 12974_2022_2417_MOESM1_ESM/Figure-S3.jpg]

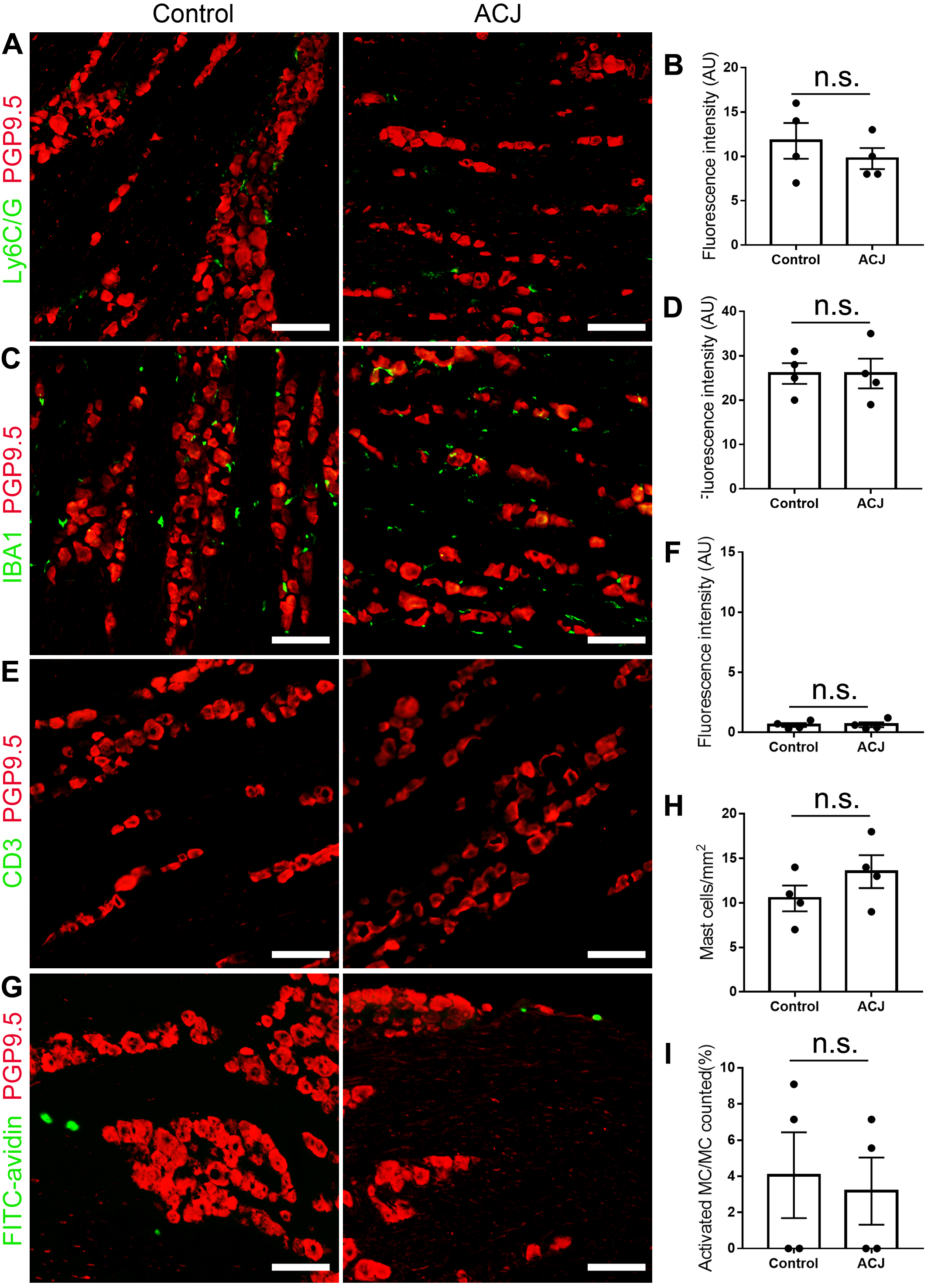

Supplement: Supplementary file 1 — Additional file 1: Figure S1. Expression of FcεRIα in MrgprA3+ pruriceptors. A, B Expression of FcεRIα in trigeminal ganglion (A) and spleen (B) of FcεRIα−/− mice. Scale bar: 50 μm. C Immunostaining by isotype IgG in the TG of WT mice. Scale bar: 50 μm. D Detection of FcεRIα by immunostaining in the TG of Mrgpra3GFP-cre mice. Scale bar: 100 μm. E Proportion of FcεRIα+ neurons among Mrgpra3+ pruriceptors. F–H Detection of FcεRIα by immunostaining in the conjunctiva of Mrgpra3GFP-Cre; ROSA26tdTomato mice. Scale bar: 20 μm. Figure S2. IgE-IC directly activates MrgprA3+ pruriceptors in vitro. A Identification of MrgprA3+ trigeminal neuron by fluorescent view. B–D Representative fluorescent view and Fura-2 ratiometric imaging of dissociated MrgprA3+ neuron (red arrow) and MrgprA3− neuron (white arrow). Scale bar = 50 μm. Figure S3. IgE-IC does not cause immune cell infiltration in mouse TG. A Mice were ocular instilled with IgE-IC (1, 10, 50 μg/ml; 5 μl), monomeric IgE (50 μg/ml; 5 μl), OVA (100 mg/ml, 5 μl) or vehicle (PBS; 5 μl), and eye-towards wiping bouts were counted over 1–12 h. The baseline was identified as recorded without any instillation. n = 8–10 mice per group; 2-way ANOVA for repeated measures followed by Bonferroni’s post hoc test. B–D Representative images of trigeminal ganglions which were taken 1 h after ocular instillation with either PBS, monomeric IgE, or IgE-IC and stained for Ly6C/G, IBA1, and CD3. Scale bar: 100 μm. E Quantification showed no significant differences in fluorescence intensity of markers among treatment groups. n = 4 per group; one-way ANOVA followed by Bonferroni’s post hoc test comparisons. F Representative images of trigeminal ganglions which were taken 1 h after ocular instillation with either PBS, monomeric IgE, or IgE-IC and stained for FITC-avidin. Scale bar: 100 μm. G Quantitative analysis of mast cells number in the TG after different treatments. n = 4 per group; one-way ANOVA followed by Bonferroni’s post hoc test compari [file 12974_2022_2417_MOESM1_ESM.zip › 12974_2022_2417_MOESM1_ESM/Figure-S4.jpg]

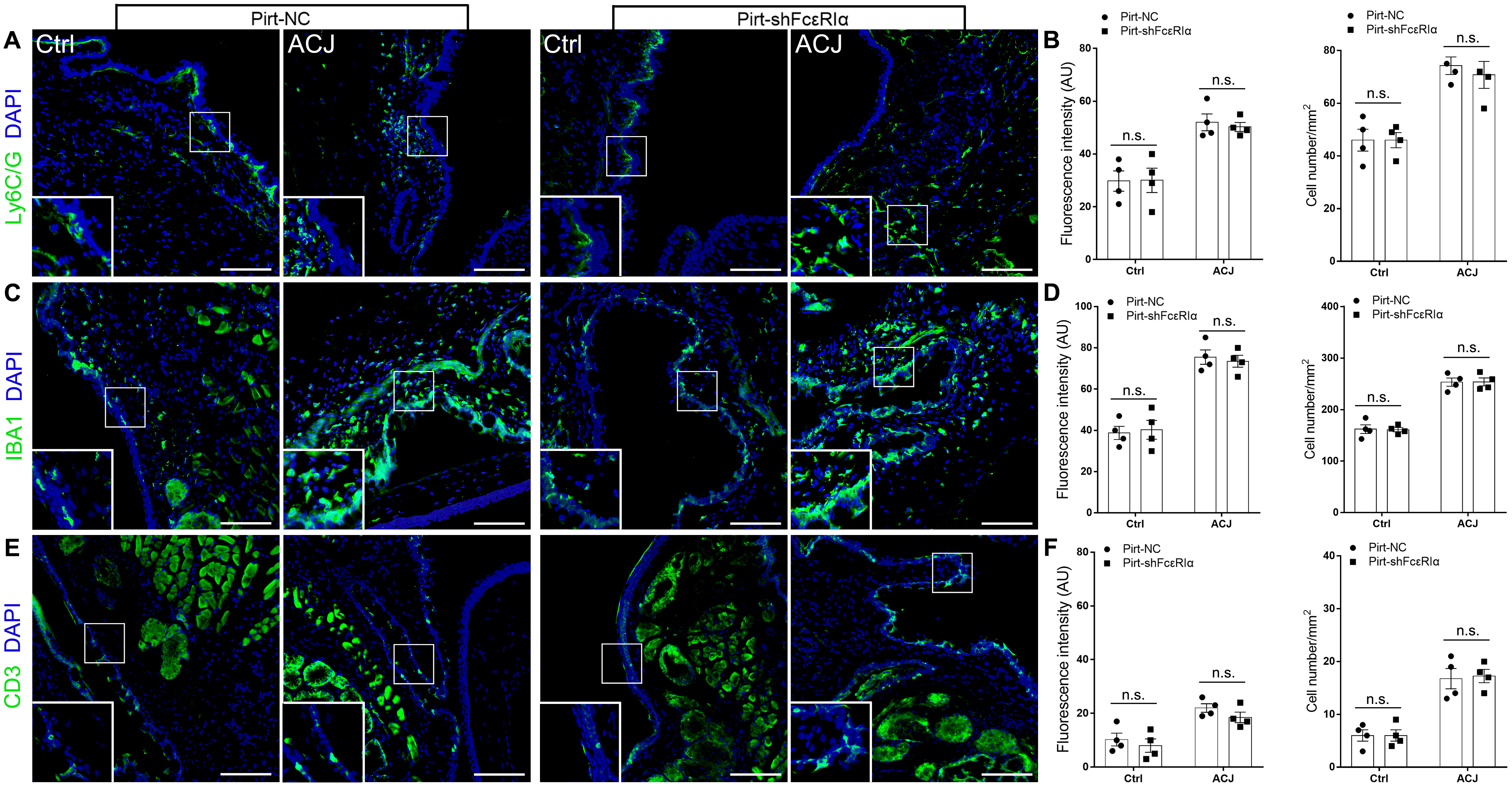

Supplement: Supplementary file 1 — Additional file 1: Figure S1. Expression of FcεRIα in MrgprA3+ pruriceptors. A, B Expression of FcεRIα in trigeminal ganglion (A) and spleen (B) of FcεRIα−/− mice. Scale bar: 50 μm. C Immunostaining by isotype IgG in the TG of WT mice. Scale bar: 50 μm. D Detection of FcεRIα by immunostaining in the TG of Mrgpra3GFP-cre mice. Scale bar: 100 μm. E Proportion of FcεRIα+ neurons among Mrgpra3+ pruriceptors. F–H Detection of FcεRIα by immunostaining in the conjunctiva of Mrgpra3GFP-Cre; ROSA26tdTomato mice. Scale bar: 20 μm. Figure S2. IgE-IC directly activates MrgprA3+ pruriceptors in vitro. A Identification of MrgprA3+ trigeminal neuron by fluorescent view. B–D Representative fluorescent view and Fura-2 ratiometric imaging of dissociated MrgprA3+ neuron (red arrow) and MrgprA3− neuron (white arrow). Scale bar = 50 μm. Figure S3. IgE-IC does not cause immune cell infiltration in mouse TG. A Mice were ocular instilled with IgE-IC (1, 10, 50 μg/ml; 5 μl), monomeric IgE (50 μg/ml; 5 μl), OVA (100 mg/ml, 5 μl) or vehicle (PBS; 5 μl), and eye-towards wiping bouts were counted over 1–12 h. The baseline was identified as recorded without any instillation. n = 8–10 mice per group; 2-way ANOVA for repeated measures followed by Bonferroni’s post hoc test. B–D Representative images of trigeminal ganglions which were taken 1 h after ocular instillation with either PBS, monomeric IgE, or IgE-IC and stained for Ly6C/G, IBA1, and CD3. Scale bar: 100 μm. E Quantification showed no significant differences in fluorescence intensity of markers among treatment groups. n = 4 per group; one-way ANOVA followed by Bonferroni’s post hoc test comparisons. F Representative images of trigeminal ganglions which were taken 1 h after ocular instillation with either PBS, monomeric IgE, or IgE-IC and stained for FITC-avidin. Scale bar: 100 μm. G Quantitative analysis of mast cells number in the TG after different treatments. n = 4 per group; one-way ANOVA followed by Bonferroni’s post hoc test compari [file 12974_2022_2417_MOESM1_ESM.zip › 12974_2022_2417_MOESM1_ESM/Figure-S5.jpg]

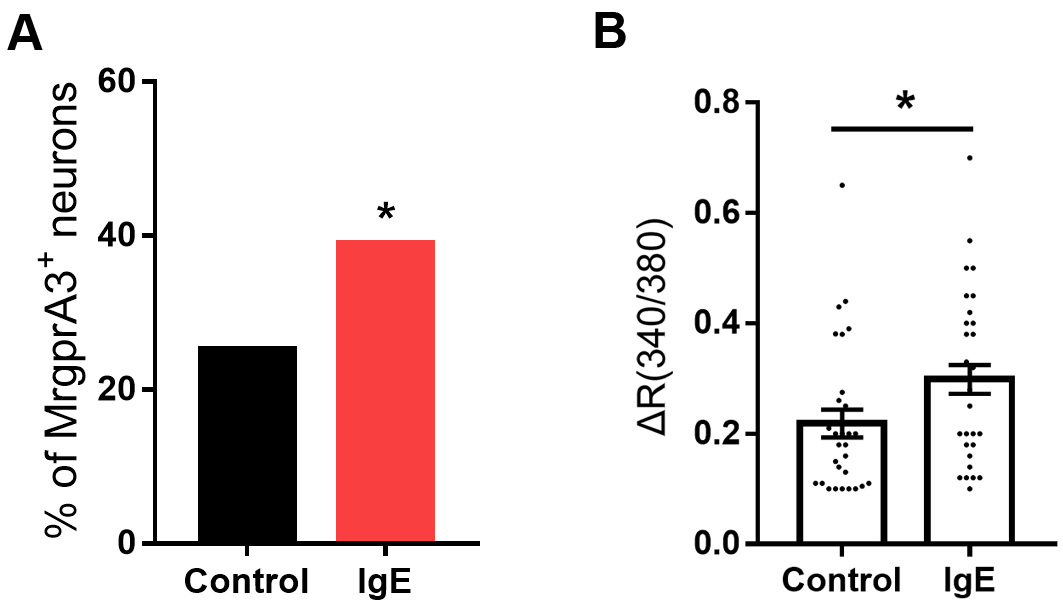

Supplement: Supplementary file 1 — Additional file 1: Figure S1. Expression of FcεRIα in MrgprA3+ pruriceptors. A, B Expression of FcεRIα in trigeminal ganglion (A) and spleen (B) of FcεRIα−/− mice. Scale bar: 50 μm. C Immunostaining by isotype IgG in the TG of WT mice. Scale bar: 50 μm. D Detection of FcεRIα by immunostaining in the TG of Mrgpra3GFP-cre mice. Scale bar: 100 μm. E Proportion of FcεRIα+ neurons among Mrgpra3+ pruriceptors. F–H Detection of FcεRIα by immunostaining in the conjunctiva of Mrgpra3GFP-Cre; ROSA26tdTomato mice. Scale bar: 20 μm. Figure S2. IgE-IC directly activates MrgprA3+ pruriceptors in vitro. A Identification of MrgprA3+ trigeminal neuron by fluorescent view. B–D Representative fluorescent view and Fura-2 ratiometric imaging of dissociated MrgprA3+ neuron (red arrow) and MrgprA3− neuron (white arrow). Scale bar = 50 μm. Figure S3. IgE-IC does not cause immune cell infiltration in mouse TG. A Mice were ocular instilled with IgE-IC (1, 10, 50 μg/ml; 5 μl), monomeric IgE (50 μg/ml; 5 μl), OVA (100 mg/ml, 5 μl) or vehicle (PBS; 5 μl), and eye-towards wiping bouts were counted over 1–12 h. The baseline was identified as recorded without any instillation. n = 8–10 mice per group; 2-way ANOVA for repeated measures followed by Bonferroni’s post hoc test. B–D Representative images of trigeminal ganglions which were taken 1 h after ocular instillation with either PBS, monomeric IgE, or IgE-IC and stained for Ly6C/G, IBA1, and CD3. Scale bar: 100 μm. E Quantification showed no significant differences in fluorescence intensity of markers among treatment groups. n = 4 per group; one-way ANOVA followed by Bonferroni’s post hoc test comparisons. F Representative images of trigeminal ganglions which were taken 1 h after ocular instillation with either PBS, monomeric IgE, or IgE-IC and stained for FITC-avidin. Scale bar: 100 μm. G Quantitative analysis of mast cells number in the TG after different treatments. n = 4 per group; one-way ANOVA followed by Bonferroni’s post hoc test compari [file 12974_2022_2417_MOESM1_ESM.zip › 12974_2022_2417_MOESM1_ESM/Figure-S6.jpg]

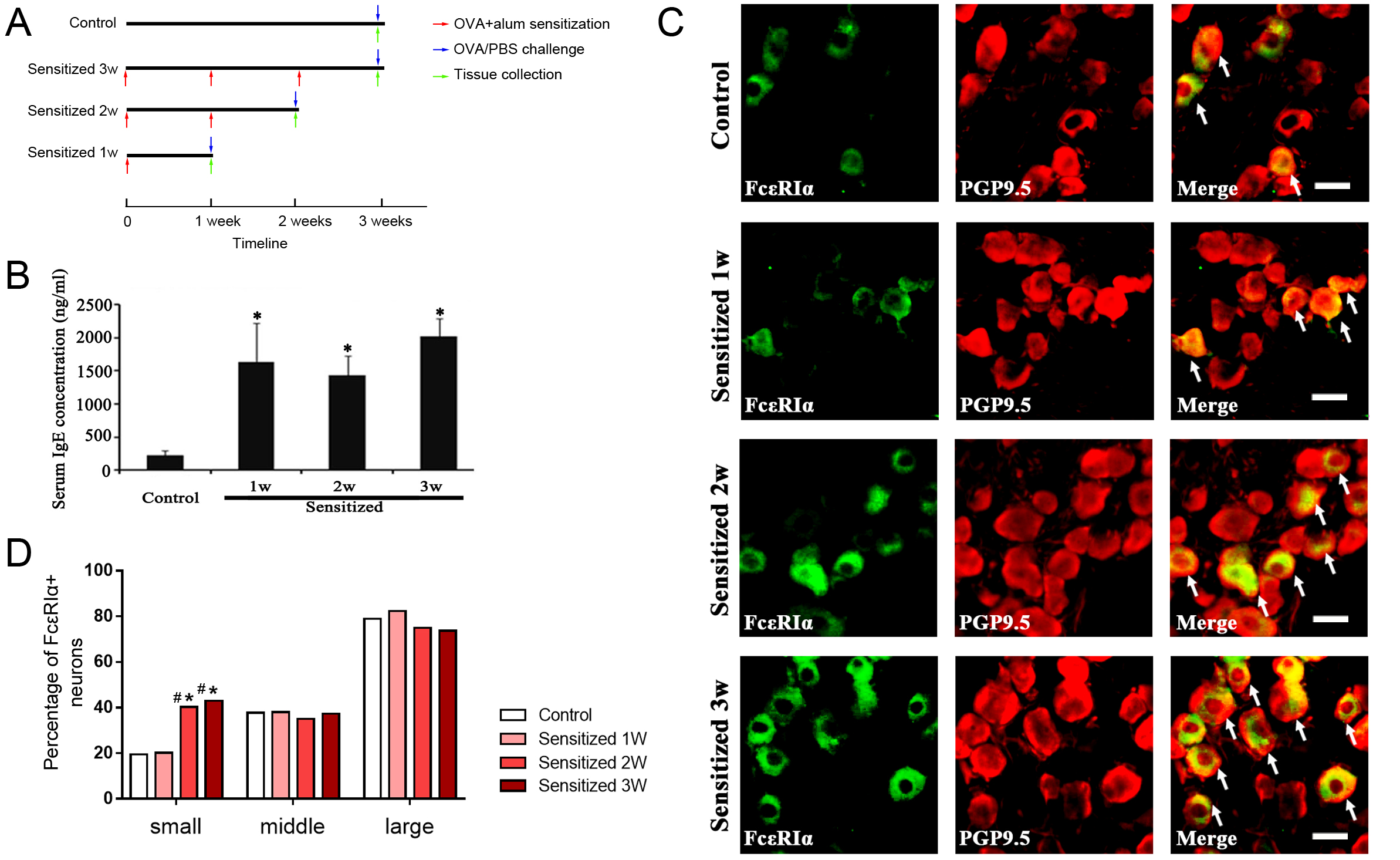

Supplement: Supplementary file 1 — Additional file 1: Figure S1. Expression of FcεRIα in MrgprA3+ pruriceptors. A, B Expression of FcεRIα in trigeminal ganglion (A) and spleen (B) of FcεRIα−/− mice. Scale bar: 50 μm. C Immunostaining by isotype IgG in the TG of WT mice. Scale bar: 50 μm. D Detection of FcεRIα by immunostaining in the TG of Mrgpra3GFP-cre mice. Scale bar: 100 μm. E Proportion of FcεRIα+ neurons among Mrgpra3+ pruriceptors. F–H Detection of FcεRIα by immunostaining in the conjunctiva of Mrgpra3GFP-Cre; ROSA26tdTomato mice. Scale bar: 20 μm. Figure S2. IgE-IC directly activates MrgprA3+ pruriceptors in vitro. A Identification of MrgprA3+ trigeminal neuron by fluorescent view. B–D Representative fluorescent view and Fura-2 ratiometric imaging of dissociated MrgprA3+ neuron (red arrow) and MrgprA3− neuron (white arrow). Scale bar = 50 μm. Figure S3. IgE-IC does not cause immune cell infiltration in mouse TG. A Mice were ocular instilled with IgE-IC (1, 10, 50 μg/ml; 5 μl), monomeric IgE (50 μg/ml; 5 μl), OVA (100 mg/ml, 5 μl) or vehicle (PBS; 5 μl), and eye-towards wiping bouts were counted over 1–12 h. The baseline was identified as recorded without any instillation. n = 8–10 mice per group; 2-way ANOVA for repeated measures followed by Bonferroni’s post hoc test. B–D Representative images of trigeminal ganglions which were taken 1 h after ocular instillation with either PBS, monomeric IgE, or IgE-IC and stained for Ly6C/G, IBA1, and CD3. Scale bar: 100 μm. E Quantification showed no significant differences in fluorescence intensity of markers among treatment groups. n = 4 per group; one-way ANOVA followed by Bonferroni’s post hoc test comparisons. F Representative images of trigeminal ganglions which were taken 1 h after ocular instillation with either PBS, monomeric IgE, or IgE-IC and stained for FITC-avidin. Scale bar: 100 μm. G Quantitative analysis of mast cells number in the TG after different treatments. n = 4 per group; one-way ANOVA followed by Bonferroni’s post hoc test compari [file 12974_2022_2417_MOESM1_ESM.zip › 12974_2022_2417_MOESM1_ESM/Figure-S7.jpg]
